# Discovering gene annotations in biomedical text databases

**DOI:** 10.1186/1471-2105-9-143

**Published:** 2008-03-06

**Authors:** Ali Cakmak, Gultekin Ozsoyoglu

**Affiliations:** 1 Department of Electrical Engineering and Computer Science, Case Western Reserve University, 10900 Euclid Ave, Cleveland, OH, USA

## Abstract

**Background:**

Genes and gene products are frequently annotated with Gene Ontology concepts based on the evidence provided in genomics articles. Manually locating and curating information about a genomic entity from the biomedical literature requires vast amounts of human effort. Hence, there is clearly a need forautomated computational tools to annotate the genes and gene products with Gene Ontology concepts by computationally capturing the related knowledge embedded in textual data.

**Results:**

In this article, we present an automated genomic entity annotation system, GEANN, which extracts information about the characteristics of genes and gene products in article abstracts from PubMed, and translates the discoveredknowledge into Gene Ontology (GO) concepts, a widely-used standardized vocabulary of genomic traits. GEANN utilizes textual "extraction patterns", and a semantic matching framework to locate phrases matching to a pattern and produce Gene Ontology annotations for genes and gene products.

In our experiments, GEANN has reached to the precision level of 78% at therecall level of 61%. On a select set of Gene Ontology concepts, GEANN either outperforms or is comparable to two other automated annotation studies. Use of WordNet for semantic pattern matching improves the precision and recall by 24% and 15%, respectively, and the improvement due to semantic pattern matching becomes more apparent as the Gene Ontology terms become more general.

**Conclusion:**

GEANN is useful for two distinct purposes: (i) automating the annotation of genomic entities with Gene Ontology concepts, and (ii) providing existing annotations with additional "evidence articles" from the literature. The use of textual extraction patterns that are constructed based on the existing annotations achieve high precision. The semantic pattern matching framework provides a more flexible pattern matching scheme with respect to "exactmatching" with the advantage of locating approximate pattern occurrences with similar semantics. Relatively low recall performance of our pattern-based approach may be enhanced either by employing a probabilistic annotation framework based on the annotation neighbourhoods in textual data, or, alternatively, the statistical enrichment threshold may be adjusted to lower values for applications that put more value on achieving higher recall values.

## Background

The number of published molecular biology and genomics research articles has been increasing at a fast rate. Advancements in computational methods expediting the predictions of thousands of genes have generated high volumes of biological data. In addition, with the advent of microarray technology, it is now possible to observe the expression profiles for thousands of genes simultaneously. Consequently, introduction of all these technologies has resulted in remarkable increases in the produced and published data.

Currently, biological knowledge recorded in textual documents is not readily available for computerized analysis. And, the current practice of manual curation of text documents requires enormous human resources. Hence, there is a need for automated computational tools to extract useful information from textual data.

The computationally extracted knowledge needs to be transformed into a form that can both be analyzed by computers and is readable by humans. To this end, different fields have developed various ontologies in an effort to define a standard vocabulary of each field. In the context of genomics, Gene Ontology (GO) [1] is proposed, continuously maintained, and used as a standardized vocabulary to express the traits of genomic entities, i.e., genes and proteins. GO consists of three subontologies, namely *molecular function, biological process and cellular component*, and contains around 20,000 concepts which are organized in a hierarchy.

Presently, GO annotations are either manually curated from the literature or computationally created (e.g., using protein domains [2]). Most of the current computational annotations are not reliable as they are mostly based on sequence homology, and high sequence similarity does not necessarily indicate GO functional coupling. The most reliable literature-based GO annotations of genes and gene products are created by biologists manually reading related papers and determining the proper GO concepts to be assigned to the corresponding genes. Usually, each annotation is accompanied by an article (or a set of articles) which is known as an *evidence article*. An evidence article for an annotation usually discusses or refers to a specific gene trait that leads to the corresponding annotation. For a GO concept *g*, the *evidence article set of g *contains all the articles that are referenced as evidence articles for the existing annotations of genes with *g*.

In this work, we focus on information extraction from biomedical publications in terms of GO concept annotations. We present a gene annotation system, called GEANN, that allows for

• Automated extraction of knowledge about various traits of genomic entities from unstructured textual data; and

• Annotating genes and proteins with appropriate concepts from GO, based on the extracted knowledge.

GEANN utilizes the existing GO concept evidence articles to construct textual extraction patterns for each GO concept. The extraction patterns are flexible in that GEANN employs semantic matching of phrases by utilizing WordNet [3]. WordNet is a repository of hierarchically organized English words that are related to other words via manually created relations like hyponyms, meronyms, and so on.

The extracted pattern set is further enriched by employing *pattern crosswalks *which involves the creation of new patterns via combining patterns with overlapping components into larger patterns. GEANN then searches PubMed publication abstracts for matches to the patterns of genomic entities, and uses the located matches to annotate genomic entities with GO concepts.

In this article, we evaluate GEANN's annotation accuracy over 114 GO concepts, where GEANN has reached to 78% precision at 61% recall on the average. GEANN is being developed as part of PathCase [4] genomic pathways database, a web-accessible bioinformatics tool for storing, visualizing and querying pathways and the associated genomic entities that take role in pathways. GEANN will be integrated into the PathCase system to provide users with newly discovered annotations and the corresponding PubMed articles leading to those annotations. In general, GEANN is used in two distinct ways:

(a) Expedite and automate the annotation of genomic entities by GO concepts with evidence extracted from scientific articles, and

(b) Enrich existing annotations with additional supporting evidences from the literature.

GEANN works at the phrase and sentence level over all PubMed abstracts. In comparison, other approaches [5,6] annotate genes over manually-assigned *reference articles *of a gene into GO concepts. Reference article set of a gene consists of articles that discuss various properties of the gene, and is selected by biologists independent of GO. Note that only well-known genes have a reference article set while majority of genes are not associated with a reference article set, restricting the applicability of other techniques. Unlike other systems [5,6], GEANN does not require the existence of a reference article set associated with each gene. Moreover, it can also provide annotation evidences at a finer phrase-level granularity rather than at the document-level. And, yet it achieves a better or comparable performance in comparison with the other systems.

There are many studies in text mining [7-10], gene annotation mining[5,6,11-13], and question answering [14-18] literature that can be considered as related [see Additional file 1 (Section 5) for a detailed discussion of these works].

In our experimental evaluation, we use scientific articles from PubMed database [19] to train and evaluate the performance of GEANN. PubMed is a digital library of over 14 million articles containing article titles and abstracts, and provides links to full texts of articles for some of the entries. Note that our approach is directly extendible to full-text of publications. However, most of the full text article access links require subscriptions to publishers' web site, hence, are not readily available for public use. Thus, we have designed and tested GEANN to work on publicly available article abstracts (see our prototype interface PubMed Abstracts Full-Text Search [20] that will be extended with GEANN).

A preliminary version [21] of this study was presented at Pacific Symposium on Biocomputing (January 2007). In this article, we extend the conference version with new content and much more rigorous experimentation on a larger data set. More specifically, with the goal of independent reproducibility, the content has been completely revamped with algorithm sketches and additional explanations at each section. We discuss and evaluate two methods to enhance the recall of the pattern-based approach to provide alternative options for applications that put higher emphasis on getting higher recall values. Pattern scoring has been completely revised, and a more intuitive statistical enrichment-based scoring scheme has been incorporated. In addition, we study the application of two different semantic similarity computation schemes, namely, edge distance-based and information content-based measures in the scope of GEANN. Furthermore, the experimental data set is extended to 114 GO terms in contrast to the previously used smaller data set of 40 GO terms in the conference version. New experimental studies are carried out: (i) Evaluation of the performance improvement due to the use semantic matching, (ii) A study of how the use of different semantic similarity computation methods influences the performance, (iii) Evaluation of the performance at different enrichment threshold values, (iv) Assessment of the performance of a probabilistic ordered-pair-based annotation framework. Finally, the related work section has been extended, and a discussion section on future directions to improve GEANN has been added.

The article is organized as follows. In the next "Methods" section, we first discuss the pattern construction process, where we present (a) the significant term discovery, (b) the pattern construction, and (c) the scoring scheme used for patterns. Next, in the Methods section, we present the second phase of GEANN, namely, the semantic pattern matching framework. Then, in the Results and Discussion section, we present an extensive experimental evaluation of our methodology and discussion of the results. More specifically, we perform a precision/recall analysis to evaluate the overall accuracy of GEANN as well as its accuracy in three distinct subontologies of GO, namely, biological process, molecular function, and cellular location. Furthermore, we compare GEANN to two other related work on a select set of GO concepts. In addition, we assess the impact of using semantic matching versus syntactic matching, and compare different semantic similarity algorithms. Finally, we discuss two alternative methods to obtain high recall values since, in some application areas, higher recall may be preferred to higher precision. More specifically, we propose a simple probabilistic annotation framework, and evaluate its performance. Next, we evaluate the accuracy, and particularly the recall, of GEANN at different significance thresholds, and then we conclude.

## Methods

In this section, we present the algorithms that we have developed within the GEANN annotation framework [see Additional file 1 (Section 1) for an illustrated overview of our approach].

### I. Pattern Discovery

Patterns are constructed based on significant terms and phrases that are associated with a GO concept. Hence, we first describe how we compute significant terms and phrases. Then, we elaborate on pattern construction and scoring.

#### Computing Significant Terms

##### Motivation

Some terms or a sequence of terms (i.e., phrases) may appear frequently throughout the abstracts of a GO concept's evidence article set. For instance, *RNA polymerase II *which is described as the machinery performing elongation of RNA in eukaryotes appears in almost all evidence articles associated with the GO concept "positive transcription elongation factor activity". Hence, intuitively, such kind of frequent occurrences within the evidence article set of a specific GO concept should be marked as a stronger indicator for a possible annotation. On the other hand, terms that are common to almost all abstracts (e.g., "cell") should be excluded from an significant term list.

##### Approach

Given (i) a GO concept C, (ii) the set Ann(C) of gene annotations by C, (iii) a database of article abstracts (PubMed in our case), and (iv) a threshold value, GEANN computes the set of significant terms for C [see Additional file 1 (Section 2.1) for a formal algorithm sketch]. For each term t that appears in an evidence article of the input GO concept C, the algorithm simply computes the number of evidence articles (tf) that are associated with C, and that contain t, as well as the number of articles (idf) that contain t in the whole input article database. Then, a simple *statistical enrichment score *is computed (as defined in the next paragraph). The terms with enrichment scores below the input threshold are excluded from the significant term list for C. Moreover, the terms that constitute the name of a GO concept that is being processed are by default considered to be significant terms.

**Definition ***(Statistical Enrichment Score)*. *Given a GO concept g, a term or phrase t, let D be the set of all articles in a database, and E be the set of evidence articles for g where E ⊆ D. Then, the statistical enrichment score Enrichment_Score(t, E) of term t in E is the ratio of t's frequency in E to the frequency of t in D. That is, EnrichmentScore(t, E, D) = f(t, E)/f(t, D) where f(t, E) and f(t, D) are the frequencies of t in T and D, respectively*.

One can use various methods to obtain the significant terms of a GO concept. For instance, correlation mining can be employed by constructing contingency matrices [22] for each article-term pair, and selecting the terms that are highly correlated with the articles of an input GO term C based on a statistical test. Alternatively, random-walk networks [23] can be constructed out of the terms appearing in evidence articles, and significant terms can be computed out of frequent word sequences that are obtained through random-walks. Since the evidence article sets of GO concepts are most of the time very small, to keep the methodology less complicated, we adopted a frequency-based approach.

Rather than using the above statistical enrichment score, we could simply use the frequency of a term in the evidence article set (i.e., its support value) as the basis of determining whether the term is a significant term or not by simply eliminating the term if its support is insufficient (i.e., below a support threshold). One of the issues that one needs to deal with in this method is setting a threshold to be used for deciding whether a given term should be included into the significant term set. Having a strict threshold sometimes results in an empty significant term set for some GO concepts, while some other GO concepts may have large sets of significant terms, not all of which may be beneficial. The main cause of such occurrences is the large variances in the size of the evidence article set available for each GO concept. For instance, assume that the significant term frequency threshold is set to 50%, i.e., for a term to be considered significant, it should appear in half of the evidence articles. For a GO concept with 10 evidence articles, terms that appear in five of them will be considered significant. On the other hand, for a GO concept with 50 training articles, terms that appear in 20 of them will be excluded from the significant term set. And, intuitively, as the size of the evidence article set increases, the possibility of detecting terms that appear in 50% of the evidence articles will decrease, in comparison to an evidence article set of smaller size. In order to get around this problem, we employ the above statistical enrichment measure to filter out the terms and phrases that are not sufficiently discriminative within the evidence article set. In this way, the problem of missing (due to the high support threshold) terms that appear in relatively low frequencies in both the evidence article set of the input GO concept C, and the input article database, but that may still be discriminative as its frequency in the evidence article set is substantially higher than its frequency in the whole article database. The effects of different statistical enrichment scores on the final accuracy are analyzed in the last section.

#### Computing Significant Phrases

A *significant phrase *is considered to be an ordered list of significant terms. Significant phrases are constructed out of significant terms through a procedure similar to the Apriori algorithm [24]. Given (i) a GO concept C, (ii) the set Ann(C) of gene annotations by C, (iii) a database of article abstracts (PubMed in our case), (iv) a threshold value, and (v) the significant term set S(C) for C, GEANN computes the set of significant phrases for C [see Additional file 1 (Section 2.2) for a formal algorithm sketch]. The algorithm creates length-(k+1) candidate phrases out of *combinable *length-k phrases, where "length" refers to the number of terms in a phrase. In order for two significant phrases SP_i _and SP_j _of length-k to be *combinable *to produce a length-(k+1) candidate phrase, the last k-1 terms in SP_i _should be the same as the first k-1 terms in SP_j_. At each iteration, those candidate phrases with enrichment scores lower than the input threshold are eliminated. This procedure is repeated until no more new phrases can be created. As an example, in the first phase, each pair of terms in the significant term set S(C) are combined (i.e., in all possible orders) to create length-2 candidate phrases. Then, the statistical enrichment score is computed for each candidate phrase, and those with enrichment scores below the input threshold are eliminated. Next, each *combinable *pair of length-2 significant phrases is merged to obtain a length-3 candidate phrase, and similarly, those with insufficient enrichment scores are eliminated.

**Figure 1:**
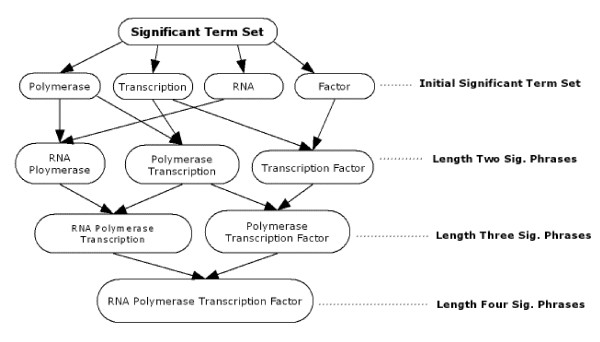
Computing Significant Phrases.

**Example 1**: Consider a set of significant term set S = {Polymerase, Transcription, RNA, Factor}. Figure 1 illustrates the level-wise significant phrase construction. At the first step, each pair of significant terms are combined to produce candidate length-2 significant phrases. Note that there are 16 distinct length-2 phrase candidates that can be constructed out of 4 significant terms. In order to avoid a complicated illustration, in Figure 1, we only show the candidates that have passed the enrichment threshold.

#### Constructing Patterns

In GEANN, the identifying elements (i.e., significant terms and phrases) of a GO concept are considered as representations of the GO concept in textual data. And, the terms surrounding the identifying elements are regarded as *auxiliary descriptors *of the GO concept represented by the pattern. In other words, a pattern represents an abstraction which encompasses the identifying elements and the auxiliary descriptors together in a structured manner. Hence, a *regular pattern*, the most basic form of patterns, is organized as a 3-tuple: {LEFT} <MIDDLE> {RIGHT} where each element of the 3-tuple corresponds to a set or sequence of words. <MIDDLE> element is an ordered sequence of *significant terms *(*identifying elements*) whereas {LEFT} and {RIGHT} elements correspond to word sets (i.e., bags of words) that occur in training articles around significant terms (*auxiliary descriptors*) where the number of terms to be considered in the elements of {LEFT} and {RIGHT} is adjusted by a *window *size.

Each word or phrase in the significant term set leads to the creation of a *pattern template*. A pattern template can be considered as a blueprint of a pattern family which specifies the middle element shared by all the members of the family. Hence, a pattern is an instance of a pattern template, and the pattern template can lead to several patterns with a common middle element, but (possibly) different left or right elements. Once a pattern template is created from a significant term or a phrase, in the training article abstracts, for each different surrounding word group that occurs around the significant term/phrase, a new instance of the pattern template, i.e., a pattern, is created. We give an example.

**Example 2**: Two of the patterns that are created from the pattern template {LEFT} <*rna polymerase II*> {RIGHT} are listed below, where *rna polymerase II *is found to be a significant term for the GO concept *positive transcription elongation factor*. {LEFT} and {RIGHT} tuples are instantiated from the surrounding words that appear before or after the significant term in the text, where the window size is set to three.

{increase catalytic rate}<rna polymerase II>{transcription suppressing transient}

{proteins regulation transcription}<rna polymerase II>{initiated search proteins}

Note that, to accommodate different forms of the same word, during the preprocessing stage, all the words are stemmed [25], and stopwords are eliminated [26]; but, for readability purposes, the words are shown in their original forms in the above example.

[see Additional file 1 (Section 2.3) for a formal algorithm sketch to construct regular patterns]. Given (i) a GO concept C for which the patterns will be extracted, (ii) C's annotation set with corresponding evidence articles, (iii) significant terms and phrases that are computed for C in the previous step, and (iv) a WindowSize value, the algorithm returns the set of all regular patterns for C. More specifically, for each significant term or phrase TP, first, a pattern template is created. Then, for each occurrence of TP in each evidence article of C, an instance of the pattern template is created where the middle tuple consists of the phrase TP, the left tuple is assigned to the first *WindowSize *words preceding TP in C, and the right tuple is assigned to the first *WindowSize *words following TP in C. Patterns are not allowed to span multiple sentences. Thus, if there are fewer words following or preceding TP in a sentence, then the size of the left and right tuples may be smaller than the WindowSize parameter. Finally, the constructed patterns are returned as the output.

#### Pattern Crosswalks

Having constructed patterns based on significant terms/phrases, we extend the initial pattern set by joining regular patterns. The goal here is to create larger patterns that may better characterize a possible GO annotation embedded into textual data. Extended patterns are constructed by virtually walking from one pattern to another. Conceptually, to walk from one pattern to another, a "bridge" connecting these patterns is located. Based on the type of bridge connecting a pair of patterns, GEANN creates two different types of extended patterns, namely *side-joined *and *middle-joined *patterns. Next, we briefly explain specific extended pattern types in the order of increasing size.

##### Transitive Crosswalk

**Figure 2:**
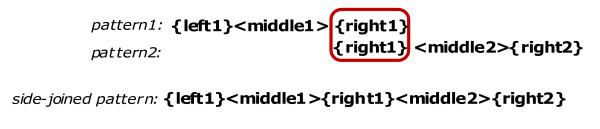
Transitive Pattern Crosswalk: Side-joined Pattern Construction.

Occasionally, created patterns may have overlapping left or right tuples. Given a pattern pair P1 = {left1}<middle1>{right1}, and P2 = {left2}<middle2>{right2}, if the right tuple of the first pattern is the same as the left tuple of the second pattern (i.e., {right1} = {left2}), then patterns P1 and P2 are merged into a 5-tuple *side-joined pattern *P3 such that P3 = {left1}<middle1>{right1}<middle2>{right2}. Side-joined pattern construction process is illustrated in Figure 2 below.

Note that, in comparison to 3-tuple regular patterns, side-joined patterns has five tuples, where <middle1> and <middle2> are sequence of words, and the remaining tuples are bags of words. Next, we give an example of a side-joined pattern that is created for the GO concept *positive transcription elongation factor *where [ge] is a tag for a genomic entity name placed by the named entity recognizer.

**Example 3**: Consider the two patterns P_1 _and P_2 _below.

P_1 _= {factor increase}<transcription rate>{RNA polymerase II}

P_2 _= {RNA polymerase II}<elongation factor>{[ge]}

Then, the side-joined pattern is:

P_3 _= {factor increase catalytic}<transcription rate>{RNA polymerase II}<elongation factor>{[ge]}

[see Additional file 1 (Section 2.4) for a formal algorithm sketch to construct side-joined patterns]. Given a set of regular patterns, the algorithm simply checks each pair of regular patterns to see if the right tuple of the first pattern is the same as the left pattern of the second pattern. If this is the case, a new 5-tuple side-joined pattern is created, and its tuples are initialized as illustrated in Figure 2.

Side-joined patterns are helpful in detecting consecutive pattern matches that partially overlap in their matches. If there exist two consecutive regular pattern matches, then such a matching should be evaluated differently than two separate matches of regular patterns as it may provide stronger evidence for the existence of a possible GO annotation in the matching region. Therefore, side-joined patterns are abstractions to capture consecutive matches.

##### Middle Crosswalk

**Figure 3:**
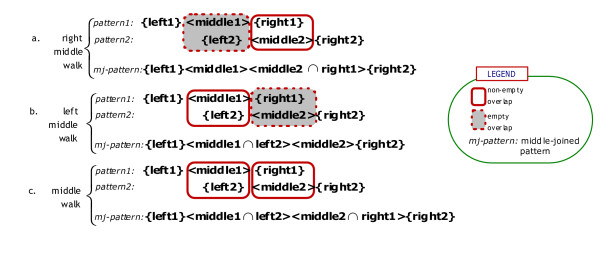
Middle Crosswalk: Middle-joined Pattern Construction.

The second type of extended patterns are constructed based on partial overlappings between the middle and side (right or left) tuples of two patterns. Since middle tuples are constructed from significant terms/phrases, a partial overlapping, that is, a subset of a middle tuple, will also be a significant term. A pattern pair P_1_={left1}<middle1>{right1} and P_2_={left2}<middle2>{right2} can be merged into a 4-tuple *middle-joined pattern *as illustrated in Figure 3.

a. right middle walk: {right1} ∩ <middle2> ≠ ∅ and <middle1> ∩ {left2} = ∅

b. left middle walk: <middle1> ∩ {left2} ≠ ∅ and {right1} ∩ <middle2> = ∅

c. middle walk: <middle1> ∩ {left2} ≠ ∅ and {right1} ∩ <middle2> ≠ ∅

In comparison to 3-tuple regular patterns, middle-joined patterns have 4 tuples: {left}<middle1><middle2>{right} where <middle1> and <middle2> are word sequences, whereas {right} and {left} are bags of words. In case (a), the first middle tuple is the intersection of {right1} and <middle2> tuples where the intersection is aligned according to the order of words in <middle2>. Case (b) is handled similarly. As for case (c), the first and the second middle tuples are subsets of <middle1> and <middle2>. Middle-joined pattern construction is illustrated in Figure 3 which is followed by an example middle-joined pattern constructed for the GO concept *positive transcription elongation factor*.

**Example 4**: Middle-joined Pattern (type (c) middle walk). Consider the two patterns P_1 _and P_2 _below where window size is three.

P_1 _= {[gp] facilitates chromatin}<transcription>{chromatin-specific elongation factor}

P_2 _= {classic inhibitor transcription}<elongation rna polymerase ii>{pol II}

Then, the resulting middle-joined pattern P_3 _is:

P_3 _= {[gp] facilitates chromatin}<transcription><elongation>{pol II}

[see Additional file 1 (Section 2.5) for a formal algorithm sketch to construct middle-joined patterns]. For each pair (P1, P2) of patterns in the input pattern set, the algorithm checks for overlaps either between middle tuple of P1 and left tuple of P2, or between right tuple of P1 and middle tuple of P2. If any overlap is found, then, according to the cases which are enumerated in Figure 3, a new 4-tuple middle-joined pattern is created, and its tuples are initialized (Figure 3).

Like side-joined patterns, middle-joined patterns capture consecutive pattern matches in textual data. In addition, since we enforce the full matching of middle tuple(s) for a valid match, partial matches to the middle tuple of a regular pattern is missed. However, middle-joined patterns accommodate consecutive *partial *matches since, by definition, their middle tuples are constructed from the intersection of a middle tuple and a side tuple of different patterns. For instance, in example 3, a partial match to P1 followed by a partial match to P2 can be accommodated by the middle-joined pattern P3. Otherwise, such a match would be missed.

#### Scoring Patterns

Pattern scores are used to assign a confidence value for a candidate annotation which is created as a result of match to a particular pattern. For scoring patterns, GEANN uses the statistical enrichment scores of significant terms/phrases as the scores of the patterns. That is, given a GO concept *g, PatternScore(P) *of a pattern *P *with a middle tuple which is constructed from a term/phrase *t *is

*PatternScore(P) *= *EnrichmentScore*(*t*, *E*, *D*) = *f*(*t*, *E*)/*f*(*t*, *D*)

where *E *is the evidence article set of *g*, and *D *is the set of all articles in the database.

Similarly, extended (i.e., side- and middle-joined) patterns are also scored based on the statistical enrichment scores of their middle tuples. However, since the extended patterns have two middle tuples, the statistical enrichment is adapted accordingly as follows. Given a side-joined or middle-joined pattern ExP with middle tuples phrases *t*_1 _and *t*_2_, and GO concept *g *with evidence article set *E, PatternScore(ExP) *is computed as

*PatternScore(ExP) *= *EnrichmentScore*(*t*_1_, *t*_2_, *E*, *D*) = *f*(*t*_1_, *t*_2_, *E*)/*f*(*t*_1_, *t*_2_, *D*)

where *f*(*t*_1_, *t*_2_, *E*) is the frequency of articles that contain both *t*_1_, *t*_2 _in E, and *f*(*t*_1_, *t*_2_, *D*) is the frequency of articles that contain both *t*_1 _and *t*_2 _in D. For middle-joined patterns *t*_1 _and *t*_2 _is required to be consecutive, while for side-joined patterns there may be up to *WindowSize *number of words between *t*_1 _and *t*_2 _to compensate for the tuple between *t*_1 _and *t*_2 _in a side-joined pattern.

The fact that we design our pattern scoring mechanism completely based on the enrichment scores of the significant phrases is closely related to the pattern construction phase. Among the elements of a pattern, the middle tuple constitutes the core of a pattern since only the middle tuple consists of phrases or terms that are determined based on frequency-based enrichment criteria. On the other hand, the remaining elements (i.e., left and right tuples) of a pattern, are directly taken from the surrounding words of significant phrases in evidence articles without being subject to any statistical selection process. Hence, middle tuples are the elements that provide the semantic connection between a pattern and the GO concept to which it belongs. Alternatively, we could use the support (i.e., the frequency among the training articles) of the significant phrase in the middle tuple. Nevertheless, enrichment score already utilizes support information (i.e., in its numerator), and further refines it by considering the global support (i.e., the frequency in the whole database) so that the influence of the patterns with significant phrases that are common to almost all articles in the database would be relatively smaller.

### Pattern Matching

Now that the patterns are obtained, the next step is searching for occurrences of patterns with the goal of predicting new annotations based on pattern matches. Given a pattern P and a article Pr, we have a match for P in Pr if (i) Pr contains the significant phrase in the middle tuple of P, and (ii) left and right tuples of P are *semantically similar *to the surrounding words around the occurrence of P's middle tuple in Pr. We require exact occurrence of P's middle tuple in Pr since the middle tuple is the core of a pattern, and it is the only element of a pattern, which is computed based on a statistical measure. And, the motivation for looking for semantic similarity rather than exact one-to-one match for side tuples is that, for instance, given a pattern P1 = "{*increase catalytic rate*}<*transcription elongation*>{*RNA polymerase II*}", we want to be able to detect phrases which give the sense that "transcription elongation" is positively affected. Hence, phrases like "*stimulates rate of transcription elongation*" or "*facilitates transcription elongation*" also constitute "semantic" matches to the above pattern.

[see Additional file 1 (Section 2.6) for a formal algorithm sketch for locating pattern matches]. Given a pattern *Pat *to be searched in a set of articles *ArticleSet*, and the GO concept that Pat belongs to, the algorithm returns a set of gene annotation predictions with their confidence scores. For each occurrence of Pat's middle tuple in an article Pr in ArticleSet, the corresponding left and right tuples are extracted from the surrounding words around the occurrence in Pr. Then, Pat's left and right tuples are compared for semantic similarity to the left and right tuples that are just extracted from Pr. We next describe the implementation of this comparison procedure function.

In order to determine the extent of semantic matching between two given sets of words WS1 and WS2, GEANN employs WordNet to check each word pair (W_i_, W_j_), where W_i_∈ WS1 and W_j _∈ WS2, if they have similar meanings. To this end, we have implemented a semantic similarity computation framework based on WordNet. Given a word pair (W_i_, W_j_), many semantic similarity measures are proposed to compute the similarity between the word pair W_i _and W_j _in different contexts [27-31]. Instead of proposing a new measure, in this study, we have implemented two of the commonly used similarity measures on WordNet. Next, we briefly describe these similarity measures and defer their performance evaluations (in our context) to the experiments section.

#### ▪ Edge Distance-based Similarity Measure

Given a taxonomy T and two nodes (representing words in WordNet) t1 and t2 in T, the most intuitive way to compute the similarity between t1 and t2 is to measure the distance between t1 and t2 in T [30,31]. As the path between t1 and t2 gets shorter, their similarity increases. That is,

Sim_edge_distance _(t1, t2) = 1/Distance(t1, t2).

**Figure 4:**
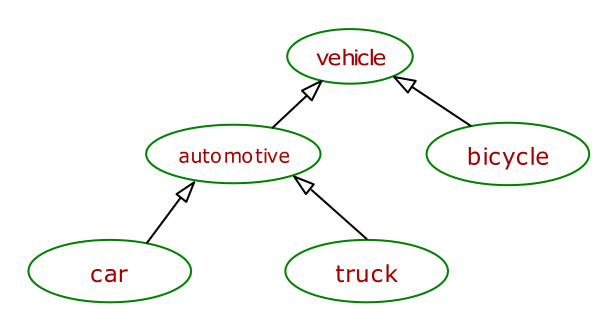
A sub-taxonomy from WordNet.

If there are multiple paths from t1 to t2, then the shortest path is selected to compute the similarity. For instance, in Figure 4, in a sub-taxonomy in the WordNet, the similarity between "car" and "truck" is Sim_edge_distance _(car, truck) = 1/2 = 0.5 while the similarity between "car" and "bicycle" is Sim_edge_distance _(car, bicycle) = 1/3 = 0.33.

#### ▪ Information Content-Based Semantic Similarity

Resnik [29] proposes a similarity measure which is based on the argument that nodes t1 and t2 in a taxonomy T are similar relative to the information shared between the two. Hence, the information content of an ancestor node t' that subsumes both t1 and t2 (i.e., t' is an ascendant of both t1 and t2) in T can be used as a measure of similarity between t1 and t2. *Information content of a node t in taxonomy T *is computed based on the occurrence probability p(t) of t in T. p(t) is the ratio of the nodes that are subsumed by t to the total number of nodes in T. Lesser occurrence probability for a node t implies a higher information content. *Information content IC(t) *of node t is quantified as -log *p*(*t*) which decreases as t gets more general in the taxonomy. As an example, the occurrence probability of node "automotive" in Figure 4 is p(automotive) = 3/5, and its information content is -log(3/5) = 0.22 whereas the information content of node "car", which is more specific, is -log(1/5) = 0.70. If there are multiple shared ancestors of t1 and t2 then the one with the highest information content is selected for similarity computation.

**Figure 5:**
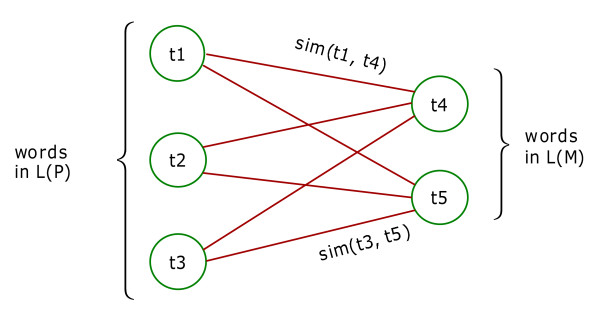
Maximum total matching of a bipartite graph.

[see Additional file 1 (Section 2.7) for a formal algorithm sketch for the implementation of matching score computation]. In order to compute the overall semantic similarity between sets of words based on the similarities between individual word pairs, we utilize an open source software library [32] which uses the Hungarian method [16] to solve the problem as follows. Given two word sets, WS1 and WS2, let n be the number of words in WS1, and m be the number of words in WS2. First, a semantic similarity matrix, R [n, m], containing each pair of words in WS1 and WS2 is built, where R [i, j] is the semantic similarity between the word at position i of WS1 and the word at position j of phrase WS2, which can be computed using either of the measures explained above. Thus, R [i, j] is also the weight of the edge from i to j. The problem of computing the semantic similarity between two sets of words WS1 and WS2 is considered as the problem of computing the maximum total matching weight of a bipartite graph [16]. This makes sense since WS1 and WS2 are disjoint in the sense that the comparisons are always made between word pairs that belong to different sets. Finally, the Hungarian method [16] is used to solve problem of computing the maximum total matching weight of a bipartite graph. For instance, consider the bipartite graph G in Figure 5 where nodes t_i _are the words from WS1 and WS2, and the weight of the edges are the semantic similarity between t_i _and t_j _where t_i _∈ WS1 and t_j _∈ WS2. The problem of computing the maximum total matching weight on G is to find a subset E' of edges in G such that no edges in E' share a node, all nodes are incident to an edge in E', and the sum of edge weights in E' is maximum.

Then, the match score of pattern P to an occurrence O of P in an article is computed as the average similarity of the semantic matching score computed for the left and the right tuples of P. That is,

(1) $$SemSim(P,O)=\frac{SemSim(P.LeftTuple,O.LeftTuple)+SemSim(P.RightTuple,O.RightTuple)}{2}$$

where P. LeftTuple is the left tuple of pattern P, and O. LeftTuple is the word set in O that matches the left tuple of P. Similarly, O. RightTuple is the word set in O that matches P. RightTuple.

The semantic similarity score returned from the WordNet evaluation is used as the base of our confidence for the match between P and O. Thus, each individual pattern match between P and O is assigned a score based on (i) the score of the pattern P, and (ii) the semantic similarity between P and O computed using WordNet (Eq. 1). That is,

(2) *MatchScore*(*P*, *O*) = *PatternScore*(*P*)*SemSim(*P*, *O*)

### Associating Pattern Matches with Genes

Having located a text occurrence O that matches the pattern P, and evaluated the match score, the next step is to decide about the genomic entity that will be associated with the match, and, hence, will get annotated with the specific GO concept the pattern belongs to. We next describe the implementation of this function. In this context, locating the corresponding gene for a candidate annotation, there are two main issues that one needs to deal with: (i) detecting terms or phrases that are gene or gene product names, and (ii) determining which one of the genes to choose, among possibly many candidates located around the matching region in the text. The first task is a particular version of the problem of developing a named entity tagger, which is an active research area in the natural language processing field. Since our focus in this study is not on developing a named entity tagger, we utilized an existing biological named entity recognizer, called Abner [33]. Abner is reported to recognize genomic entities with 68% precision and 77% recall, and it is one of the most accurate entity recognizers in terms of performance, according to experimental comparisons against similar systems [33].

Once the gene names in the text are tagged by the named entity tagger, the next task is to decide on the gene to be annotated. This task is not straightforward as there may be several gene products/genes around the matched phrase in the abstract. Thus, we need to find a mechanism to correctly recognize the genomic entity the matched occurrence O refers to. Our approach is based on a set of heuristics: we first look into the sentence containing the matching M, and choose the gene product that comes first before the matching phrase in the same sentence. If we cannot find one, then we check the part that follows the matching region in the same sentence. If there is no gene name mentioned in the same sentence, we check the previous and the following sentences, respectively.

Finally, each predicted annotation is assigned an annotation (confidence) score. The final annotation score of a gene *g *by a pattern P with occurrence O in the text is a function of both the match score of P to O (Eq. 2) and the distance of the reference to the gene in the text to O, that is

(3) *AnnotationScore*(*P*, *O*, *g*) = *MatchScore*(*P*, *O*)*F_*Distance *_(*g*, *O*, *t*, *n*)

where *F*_*Distance *_is the distance function, *t *is the distance of gene reference *g *to occurrence O in terms of the number of words between them, and *n *is the minimum number of sentences that span *g*, O, and the set of words between g and O. As an example, if *g *and O are in the same sentence, *n *= 1, and if *g *reference is in the next sentence that follows the sentence containing O then *n *= 2, and so on. Intuitively, the distance function should generate lower scores as the distance *t *increases. In addition, being located in different sentences should considerably decay the distance function value. Therefore, *F*_*Distance *_should be a monotonically decreasing function as *t *or *n *increases. In this article, we use the following heuristic distance function that conforms to the above intuitions concerning t and n.

(4) $$F_{Dis\tan ce}(g,O,t,n)=\{\begin{array}{ll} 1 & if0\leq t^{n}≺2\\ 1/log(t^{n}) & otherwise \end{array}$$

Alternative distance functions are possible as long as those alternatives are monotonically decreasing as t or n increases. While designing the above particular function, we choose to incorporate n, for instance, as an exponent of distance t as we have observed in several examples that reliability annotations decay significantly when a pattern match in a sentence is used to annotate a gene in a different sentence. In contrast, it is our observation that the impact of the distance parameter t is less severe in comparison to n. Thus, t is incorporated to affect the value of the function in a linearly inverse proportional manner.

## Results and Discussion

### Experimental Setup and Dataset

In order to evaluate the performance of GEANN, we perform experiments on annotating genes in NCBI's GenBank [34]. During the experiments, we exclude an article abstract from our testing/training dataset if it does not contain a reference to any of the gene products for which it is given as the annotation evidence since it is not possible to extract information from such abstracts. We also exclude those genes from our dataset that could not be located in the article abstract that it refers to as part of its annotation. This way, we aim to clean the noise in the data, which would not be useful to train or test the GEANN system. These kinds of exclusions usually occur for article abstracts that discuss the sequencing of whole organisms; and, hence, individual gene or protein names belonging to the sequenced organism do not usually appear in such article abstracts. In addition, each annotation in GO is accompanied by an *evidence code *which indicates how the annotation is created, i.e., how reliable it is. The least reliable annotations are the ones that have the evidence code *IEA (Inferred from Electronic Annotation) *which are computationally created, and not curated. Therefore, we exclude such annotations from our training data.

Our experiments are based on the precision-recall analysis of the predicted annotation set under several circumstances. To this end, for each case, we adopt the k-fold cross validation scheme [35] as follows: the existing, known annotation set is divided into k parts (k = 10 in our case), and (k-1) parts are used to train GEANN, and the remaining one part is used to test the trained GEANN system. The same procedure is repeated k (i.e., 10) times, each time, using a different partition for testing, and the rest of the annotation set for the training. Finally, individual experimental results are normalized, and averaged to obtain a combined result set for a given GO concept. Next, we formally define the precision and recall as well as F-value which are used as performance measures during interpretation of experimental results.

**Definition ***(Precision)*: *Given a GO concept C and the set S of predicted annotations for C, precision for C is the ratio of the number of genes in S that are correctly predicted to the total number of genes in S*.

**Definition ***(Recall)*: *Given a GO concept C and the set S of predicted annotations for C, recall for C is the ratio of the number of genes in S that are correctly predicted to the number of genes that are known to be annotated with C*.

**Definition ***(F-Value)*: *F-Value is the harmonic mean of precision and recall, and computed as*

*F-Value(Precision, Recall) *= (2 * Precision * Recall)/(Precision + Recall)

**Figure 6:**
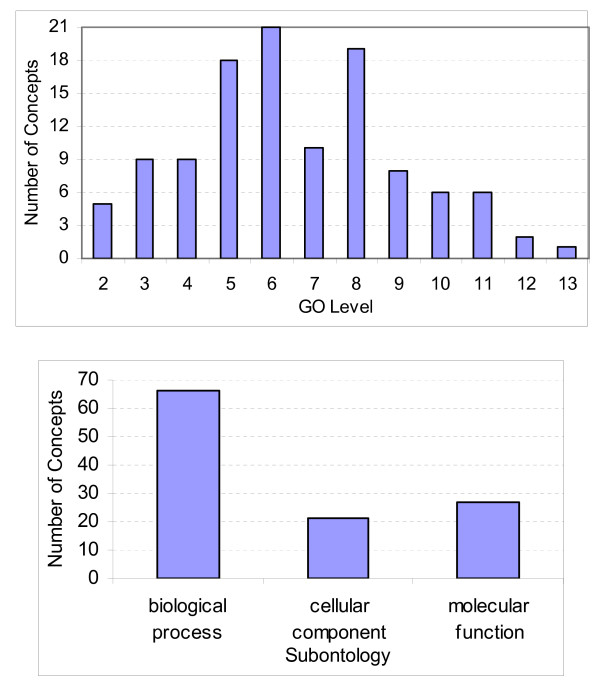
Distribution of GO concepts in the test set.

Since we perform 10-fold cross validation, for an accurate analysis, we enforce the requirement that each partition has at least three evidence articles to test during the evaluations. Hence, we make sure that each selected GO concept for experimental evaluation has at least 30 evidence articles and genes. Thus, the experimental GO concept set consists of 114 GO concepts [see Additional file 2] from all three subontologies of GO, namely, biological process, cellular component, and molecular function subontologies. Distribution of the experimental GO concepts by their corresponding GO subontology and the level of the GO hierarchy are presented in Figure 6 where the root level of each subontology is considered as level 1. 114 GO concepts had a total of 8,556 articles referenced as evidences for annotation of a total of 12,047 genes. In total, 1,751 articles and 4,690 genes were removed from the data set, which left us with 6,805 article abstracts and 7,357 genes to be used during the evaluation. On the average, each GO concept has 60 evidence articles and 65 gene annotations.

In order to approximate the word frequencies in the actual PubMed database (required to compute the statistical enrichment scores), we used a larger corpus of 150,000 article abstracts which consist of articles that are referred to in support of an annotation in GO (our training set). This corpus is only used for the calculation of statistical enrichment scores, and consists of articles that Genbank curators list as related reference articles for the genes in the Genbank database. Reference article set for each gene is part of the Genbank database, and it is publicly available [34] to download or browse online. As part of pre-processing, abstracts/titles of articles are tokenized, stopwords are removed, words are stemmed. Non-alphanumeric characters are removed, as they are not useful for extracting patterns. Each abstract/title is run through the biomedical named entity tagger Abner [33] to mark genes and gene products.

GEANN maps gene name occurrences found in PubMed article abstracts to actual gene records in GenBank. One major problem in this type of study is to determine which entities from two different data sources are really referring to the same object. The reconciliation process also known as the entity disambiguation problem [36] by itself is a separate research problem. In this study, we ignore the genomic entities annotated by GEANN and yet do not have any corresponding entry in GenBank. Furthermore, a genomic entity symbol that is annotated through the procedure we described so far may match to more than one gene or gene product in GenBank because of the shared synonyms between different genes. Thus, in this study, as a reconciliation scheme, GEANN uses three assumptions.

***A1****(Handling Shared Gene Synonyms): *Among the GenBank genes that match to the symbol being annotated, if at least one of the matched genes has the annotation involving a particular GO concept, then this annotation prediction is considered as a correct prediction (or true positive). On the other hand, if none of the genes sharing the gene symbol of the predicted annotation has a record corresponding to the particular GO concept among its GO annotations, then such results are considered as incorrect predictions or *false positives*.

***A2****(Annotating via the GO Hierarchy): *If one of the matche genes in GenBank is annotated with a descendant of the given GO concept G, then G also annotates the gene (i.e., a *true positive*) due to the *true-path rule *of GO, which states that if the child concept annotates a gene, then all its ancestor concepts also annotate that gene.

***A3****(Using Annotations in Genbank that have have no Literature Evidence): *If the predicted annotation is included in GenBank, then we consider this prediction as a *true positive *regardless of (a) its evidence code, and (b) whether it has a literature reference.

#### Experiment 1: Overall Performance

We first evaluate the overall performance of GEANN. Predicted annotations are ordered by their annotation scores. First, precision and recall values for individual GO concepts are computed by considering the top-k predictions where k is increased by one at each step until either all the annotation information available for a GO concept is located, or all the GO candidates in the predicted set are processed. Next, the precision/recall values from individual GO concept assessments are combined by taking the average of precision/recall values at each k value for top-k results.

**Figure 7:**
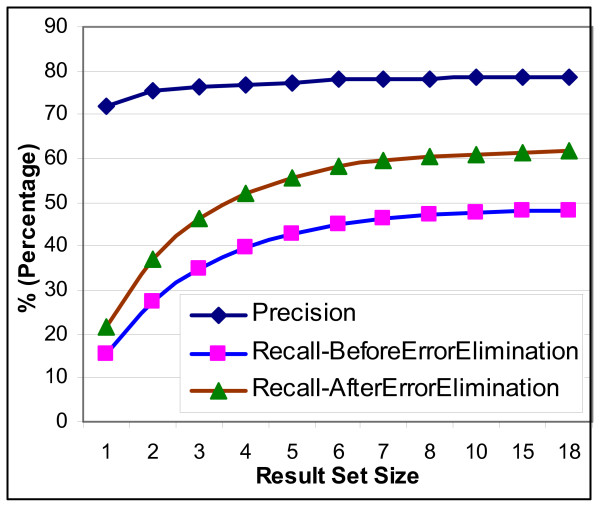
Overall System Performance & Approximate Effect of the Error due to the Named Entity Tagger.

From Figure 7 which presents the average precision/recall values that were computed for different predicted gene set sizes, we have

***Observation 1***: *GEANN yields 78% precision at 48% recall.*

Note that the accuracy of the tagging gene/gene products in the text influences the association of a pattern to a genomic entity. However, named entity taggers (NETs) also negatively affect the accuracy. In the rest of the paper, we consider this negative effect [For more details, see Additional file 1 (Section 3.1)].

#### Experiment 2: Annotation Accuracy across Different Subontologies in GO

Next, we evaluate the accuracy of GEANN across the three different subontologies of GO, namely, biological process, molecular function, and cellular location.

***Observation 3***: *In terms of precision, GEANN provides the best precision for the oncepts from cellular component (CC) and molecular function subontologies where precision is computed as 80% while biological process (BP) subontology yields the highest recall (63% at 77% precision). The fact that MF subontology provides better precision may be due to the fact that biological process concepts refer to biological pathways, and pathways are more general biological abstractions in comparison to the specific functionalities of enzyme proteins/genes, a number of which is included in each pathway*.

[see Additional file 1 (section 3.2) for more details].

#### Experiment 3: Comparative Performance Analysis with Other Systems

In this section, we compare our approach to two other studies, namely, Raychaudhuri et al. [5] and Izumitani et al. [6], that build document classifiers to label the genes with GO concepts through the documents associated with them. First, both approaches assume that a gene is associated with several articles. This is a strong assumption in that if the experts are to invest considerable time to read and associate a set of articles with a gene, then they can probably annotate the gene themselves with the appropriate GO concepts. Under this assumption, these systems are not practically applicable to automate gene annotation. Second, since both of the systems work at the document level, no direct evidence phrases are extracted from the text. Third, GEANN can also provide the matching phrases as a source of evidence rather than the whole document. In this experiment, we show that, even though GEANN does not have the strong assumptions that these systems use, and can work with much smaller sets of training data, GEANN's performance is still comparable to or better than these systems. Furthermore, GEANN handles the reconciliation of two different genomic databases based on the tagging of genes and proteins by an entity tagger (namely, ABNER), whereas the studies discussed here have no such considerations as they assume that such a mapping is already provided to their systems with some additional associated articles.

Using 12 GO concepts, Izumitani et al. compares its system to Raychaudhuri et al.'s study. To provide a comparison, our analysis in this experiment is also confined to this set of GO concepts. The following GO concepts could not be cross-validated due to their small annotation set size: *Ion homeostasis GO:0006873 (6 annotations)*, *Membrane fusion GO:0006944 (8 annotations)*. Furthermore, one of the test concepts (Biogenesis) has since become obsolete. Therefore, here we present comparative results for the remaining nine GO concepts in terms of F-values. Table 1 provides the F values for these systems and GEANN. Table 2 provides F-values in terms of the subontologies.

**Table 1:** Comparing F-Values against Izumitani et al. and Raychaudhuri et al.

GO category	**GEANN**	**Izumitani et al.**	**Raychaudhuri et al.**		
			
			**Top1**	**Top2**	**Top3**
Autophagy GO:0006914	**0.93**	0.78	0.83	0.66	0.38
Cell adhesion GO:0007155	**0.77**	0.51	0.19	0.19	0.13
Signal transduction GO:0007165	**0.77**	0.76	0.41	0.30	0.21
Response to stress GO:0006950	0.64	**0.65**	0.41	0.27	0.24
Transport GO:0006810	0.64	**0.83**	0.56	0.55	0.49
Cell Proliferation GO:0008283	0.64	**0.76**	0.00	0.03	0.06
Metabolism GO:0008152	**0.92**	0.91	0.42	0.65	0.74
Cell Death GO:0008219	**0.64**	0.58	0.07	0.06	0.02
Sporulation GO:0030435	**0.70**	0.37	0.22	0.14	0.07
*Average*	*0.74*	*0.68*	*0.40*	*0.33*	*0.25*

**Table 2:** Comparing F-Values against Izumitani et al. For GO SubOntologies

**GO Subontolgy**	**GEANN**	**Izumitani et al.**
Biological Process	0.69	0.60
Molecular Function	0.65	0.72
Cellular Location	0.69	0.58
*Average*	0.68	0.63

Table 1. **Comparing F-Values against Izumitani et al. and Raychaudhuri et al.**

Table 2. **Comparing F-Values against Izumitani et al. For GO SubOntologies**

***Observation 4***: *GEANN's performance is comparable to or better than Izumitani et al. and Raychaudhuri et al. In terms of the average F-value over the test GO concept set of size 9, GEANN outperforms both systems, and for six of the nine test concepts, GEANN performs the best*.

#### Experiment 4: Comparing Semantic Matching against Syntactic Matching

**Figure 8:**
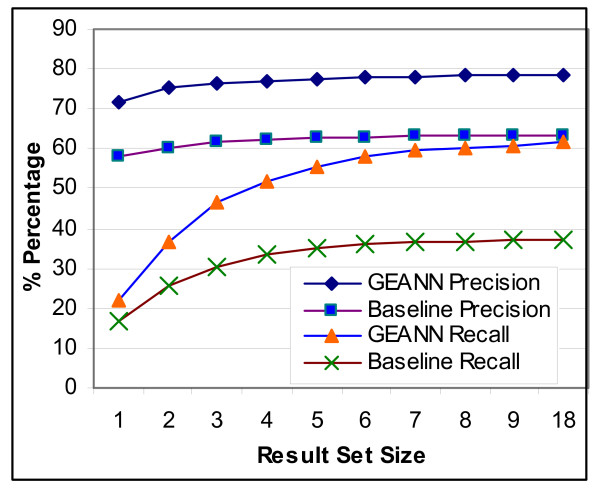
Evaluation of the improvement due to use of WordNet.

Next, we experimentally measure the improvement provided directly by the use of WordNet as the semantic similarity infrastructure. For comparison purposes, we developed a baseline methodology by replacing the semantic similarity pattern matching part in GEANN's implementation with a naive syntactical pattern matching method which recognizes only exact (i.e., word-by-word) match between a pattern and a textual phrase. All the other scoring and pattern construction mechanisms are kept the same for both the baseline system and the GEANN in order to focus on the semantic pattern matching infrastructure of GEANN. Then, we run both the baseline approach and GEANN on our experimental set of 114 GO concepts. Figure 8 provides the precision/recall values for both of the systems.

***Observation 5***: *GEANN with semantic matching outperforms the baseline approach by 24% in terms of recall and 15% in terms of the precision*.

The improvement in the accuracy is expected since not only exact matches to the side tuples of the patterns, but also approximate matches can be located and scored based on the well-studied taxonomy similarity measures and by utilizing semantic relationships between the concepts of WordNet. Table 3 lists the top-10 GO concepts which experience the most dramatic improvements in terms of their annotation accuracy in comparison to the baseline approach.

**Table 3:** Example GO concepts whose precision and Recall is improved significantly due to use of WordNet

***GO Concept***	***Recall Improvement***	***Precision Improvement***	***F-Value Improvement***
GO:0005856	55.6	88.1	68.5
GO:0001501	62.9	52.2	60.9
GO:0009887	44.0	85.7	59.4
GO:0006928	46.4	71.7	56.6
GO:0006350	42.8	73.3	54.6
GO:0005975	50.0	50.0	52.4
GO:0005489	37.8	76.2	51.8
GO:0005576	37.2	66.7	49.9
GO:0006508	35.8	68.5	48.4
GO:0008544	43.1	49.5	46.3

**Figure 9:**
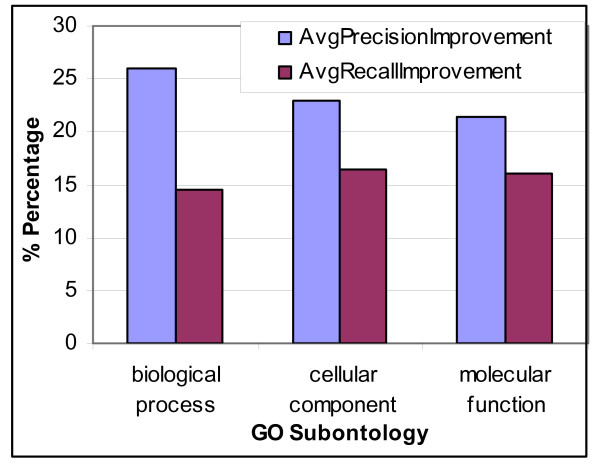
Distribution of the improvement brought by semantic similarity over different subontologies.

Next, we explore if the semantic pattern matching approach performs better for the GO concepts from a specific subontology of GO. Figure 9 shows the distribution of the improvement that GEANN brings over the baseline approach for each subontology in GO.

***Observation 6***: *Semantic pattern matching approach performs almost equally well for each subontology of GO, and the prediction accuracy improvement is more or less uniformly distributed over different subontologies*.

This indicates that semantic pattern matching approach is not specific to a particular set of GO concepts, but is effective throughout the whole GO ontology.

**Figure 10:**
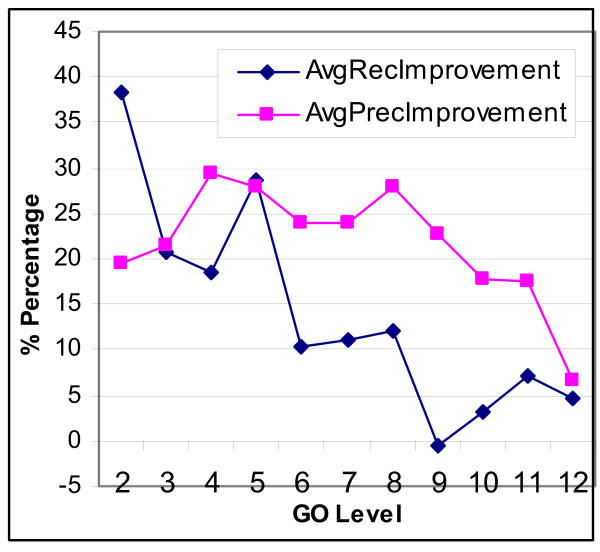
Semantic Similarity Improvement at Different Levels of GO.

Since GO is hierarchically organized, GO concepts that are closer to the root concept represent more general biological knowledge than those that are closer to the leaf levels. Hence, to see how the semantic pattern matching framework performs at different levels of GO, next we cluster the concepts by the GO level they reside, and analyze the cross-validation accuracy by changing the GO level. Figure 10 shows the change of improvement brought by GEANN over the baseline method at different GO levels.

***Observation 7***: *There is no perfect regularity in terms of changes in recall/precision improvement, as the concepts get more specific*.

***Observation 8***: *There is a general trend of decrease in both precision and recall improvement as the concepts get more specific*.

The semantic similarity measures rely on the existence of a path between the synsets (i.e., different meanings) of the words that are compared. Intuitively, as the GO level gets deeper towards the leaf level, the concepts gets more specific. Hence, the sentences describing such concepts would be more likely to include terms that are domain-specific and less likely to be found in WordNet, which narrows down the space that WordNet can be influential. In addition, since WordNet is a general purpose English word taxonomy, and is not specific to the biomedical domain, its capacity to accommodate the terms in the biology domain should not be overestimated. For instance, during our experiments, around 25% of the semantic similarity computations returned the score of zero.

#### Experiment 5: Effect of Using Alternative Semantic Similarity Measures

As there are many alternative measures that have been proposed in the literature to compute semantic similarity over the taxonomies, it is informative to explore the impact of different measures on GEANN's accuracy. Evaluation of alternative measures is by itself the main topic of many research articles [27,28,37]. In this section, our goal is by no means to provide a comprehensive study of different measures in this article's context, but to present an assessment of how the replacement of an adopted similarity measure may affect the rest of the framework. To this end, we have implemented two well-known semantic similarity measures, namely, the information content (IC)-based and edge-counting methods. We ran GEANN on the experimental set of GO concepts twice using a different measure at each run, and then compared average precision and recall of each run.

***Observation 9***: *Replacement of IC-based semantic similarity computation with edge-counting method does not cause dramatic changes on the overall accuracy of GEANN (recall for IC-based measure was just 0.4% above edge-counting method, while precision stayed the same). A couple of GO concepts had dramatic change (10%) on their either recall or precision, such occurrences were not sufficiently common to influence the overall accuracy significantly*.

The above observation is reasonable since the proposed framework here is not primarily based on the type or the nature of the adapted similarity measure. What is crucial to GEANN's success in using semantic similarity over a traditional syntactical pattern matching system is the adoption of a flexible matching system that takes advantage of semantic relationship of words, which is not always intuitively or readily available in a typical pattern matching system. Hence, the above observation confirms that (a) the adopted similarity measure is only a plug-in tool in the overall framework, (b) a particular measure is not at the core of our paradigm, (c) any of the well-known semantic similarity measures that are studied in the literature are likely to be employed by GEANN.

Table 4 – **Top-10 most affected GO concepts when the semantic similarity measure is replaced by another one**

**Table 4:** Top-10 most affected GO concepts when the semantic similarity measure is replaced by another one

***Level***	***GO Subontology***	***GO Concept***	***F-value change***
12	cellular_component	GO:0005741	-4.3
8	biological_process	GO:0016481	-4.4
5	biological_process	GO:0006955	-4.6
3	biological_process	GO:0009653	-4.7
6	biological_process	GO:0006350	-5.0
8	biological_process	GO:0007050	-6.0
3	molecular_function	GO:0005200	-6.2
8	biological_process	GO:0030036	8.1
7	molecular_function	GO:0004842	5.4
5	biological_process	GO:0008544	4.6

**Figure 11:**
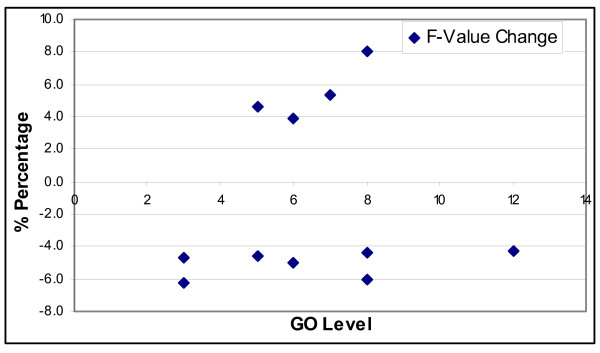
Distribution of F-value changes at different levels of GO.

Next, we further examine the small set of GO concepts that were affected by the change of the similarity measure. About 10 GO concepts experienced an F-value change greater than 4%, which are given in Table 4. Figure 11 maps the change in F-values of GO concepts to their GO levels. From this figure, there is no obvious correlation or trend that one may observe towards the relationship between F-value changes and GO-concept levels. In Table 4, among the top 10 most affected GO concepts, 7 of them belong to biological process ontology, 2 of them to molecular function, and 1 of the concepts belongs to the cellular component subontology. From this limited view of distribution, it may be tempting to look for some kind of correlation between biological process concepts and a particular semantic similarity measure. However, this may be biased as 66 of the 114 GO concepts in the experimental set are from the biological process subontology. It is not clear to us why these particular GO concepts are negatively or positively affected when the semantic similarity measure is changed from the edge-counting method to the IC-based measure. In previous studies, these two semantic similarity measures are studied in different contexts, and there is no consistent superiority of one measure to another throughout different studies reported in the literature.

### Enhancing Recall

As illustrated through the experimental results, an inherent drawback of pattern-based text mining systems is the fact that their recall performance is frequently low. In this section, we describe and evaluate two different approaches to obtain annotation predictions with high recall: (i) through a probabilistic annotation framework, and (ii) by adjusting statistical enrichment threshold value [see Additional file 1 (Section 4) for an elaborate discussion of these two approaches and the associated experimental results].

***Observation 10***: *At recall of 61% which is the maximum recall that GEANN can achieve at its maximum precision level, the probabilistic approach has a precision of 51% while GEANN has precision of 78%*.

***Observation 11***: *The probabilistic approach can reach to higher recall values (77% at the maximum) which is significantly higher than what GEANN provides (61% at the maximum)*.

***Observation 19***: *Adjusting enrichment threshold to lower values results in higher recall than the maximum recall value provided by the probabilistic approach*.

## Conclusion

In this article, we explore a method that automatically infers new GO annotations for genes and gene products from PubMed abstracts. To this end, we develop GEANN that utilizes the existing annotation information to construct textual extraction patterns characterizing an annotation with a specific GO concept. During the annotation stage, GEANN searches for phrases in PubMed abstracts that match the created patterns. Matches are scored and associated with the most proper genomic entity or a set of entities around the matching region. As the final output, GEANN lists the genes that are predicted to be annotated with a given GO concept. In our experiments, GEANN either has outperformed or is comparable to earlier automated annotation work.

[For a much more detailed discussion of future and related work see Additional file 1 (sections 5 and 6)].

## Authors' contributions

AC designed the study, drafted the manuscript and carried out the experimental studies. GO participated in its coordination and helped to draft the manuscript. All authors read and approved the final manuscript.

## Supplementary Material

Additional file 1 **SupplementaryMaterial**. This file contains detailed discussion on sections that are not included (or are briefly mentioned). More specifically, the supplementary material document contains an illustration of the overall approach, formal algorithm sketches for procedures described in the main manuscript, some additional experimental results, an elaborate comparative discussion on two alternative approaches to obtain higher recall values, and a detailed discussion on related and future work. Click here for file

Additional file 2 **Appendix 1**. This file contains the experimental GO concept set along with overall precision/recall values for each GO concept. Click here for file
