# Supplementary material for: Discovering gene annotations in biomedical text databases
Source: BMC Bioinformatics. 2008 Mar 6;9:143. doi: 10.1186/1471-2105-9-143 (PMC2335285; doi:10.1186/1471-2105-9-143)
Supplement: Additional file 1 — SupplementaryMaterial. This file contains detailed discussion on sections that are not included (or are briefly mentioned). More specifically, the supplementary material document contains an illustration of the overall approach, formal algorithm sketches for procedures described in the main manuscript, some additional experimental results, an elaborate comparative discussion on two alternative approaches to obtain higher recall values, and a detailed discussion on related and future work. [file 1471-2105-9-143-S1.pdf]

Discovering gene annotations in biomedical text databases

Supplementary Material

Ali Cakmak, Gultekin Ozsoyoglu

Department of Electrical Engineering and Computer Science,
Case Western Reserve University, 10900 Euclid Ave, Cleveland, OH, USA

1. Overview

The GEANN implementation encompasses two main phases, namely, the training and the annotation phases. Figure 1 illustrates the overall approach.

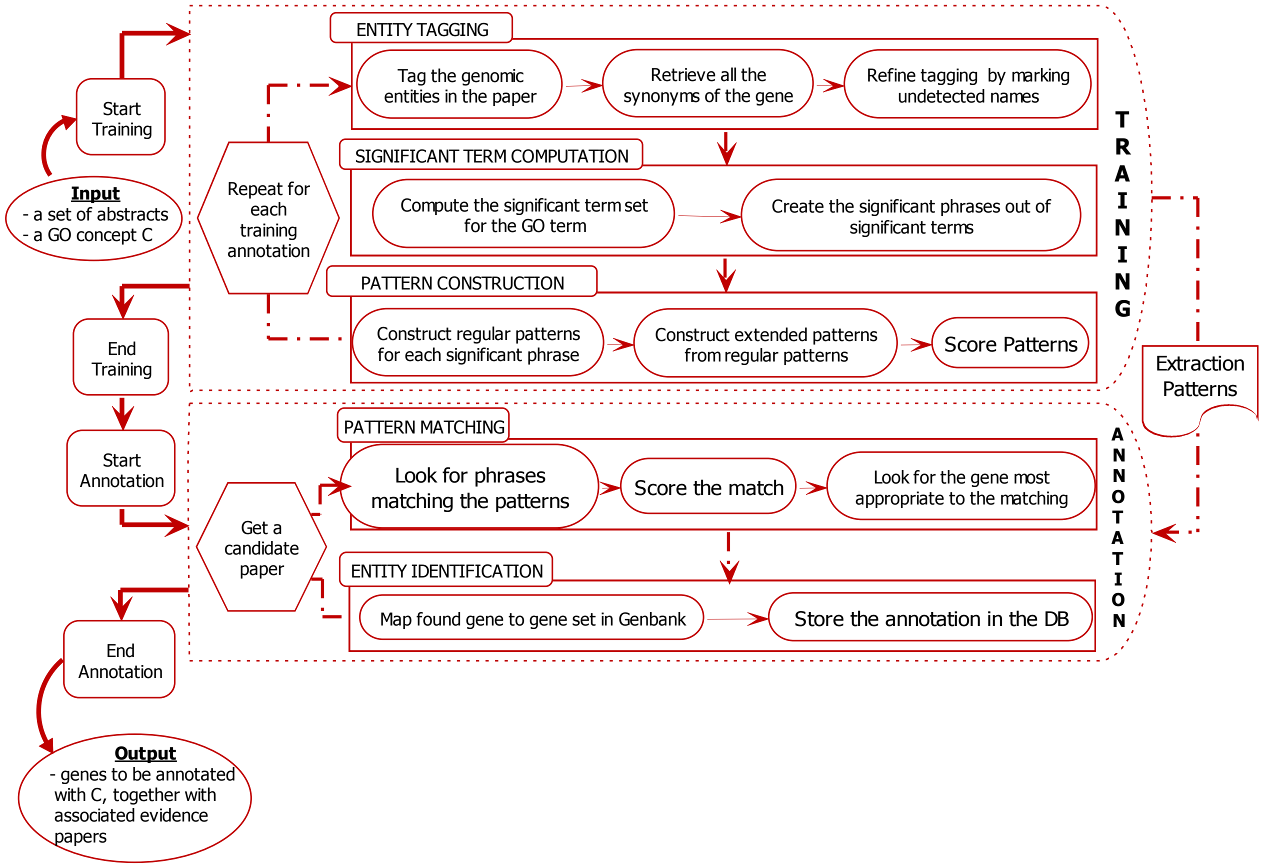

Figure 1. Steps of the process performed towards automated annotation of genomic entities

Given a GO concept C, the goal of the training phase is to construct extraction patterns characterizing a variety of indicators towards the existence of an annotation by C. In order to extract the patterns, we employ *supervised learning*. Evidence papers of the existing annotations by C are used as the training data. The first step in the training phase is the *tagging of genomic entities* that appear in a given training paper. Tagging is further refined by using the synonyms of genes to locate additional gene symbols/names that are missed during the initial automated entity tagging stage. Synonyms of a gene can be obtained from any gene database, e.g. GenBank [34]. Then, we compute *significant terms* for C. Significant terms are determined through a *statistical enrichment score* which is a ratio between a term t's frequency among the training papers and t's frequency among the remaining papers in the database. Significant term set for a GO concept C also includes those words that appear in C's name.

Next, *significant phrases* for C are created by combining significant terms of C through a procedure like the Apriori algorithm [24]. More specifically, we first combine each pair of significant terms to obtain two-term phrases (i.e., phrases that contain two terms). Then, we compute the enrichment score of a two-term phrase and eliminate the ones whose enrichment scores are below a certain threshold. Next, we construct three-term phrases by combining two-term phrases p1 and p2 where p1 and p2 overlaps except for the first term in p1 and the last term

in p2. Similarly, after eliminating three-term phrases with enrichment scores below the threshold, we construct four-term phrases by combining three-term phrases and so on until no new phrases can be created. Once the *identifying elements* (i.e., significant terms and phrases) of C are extracted, patterns of C are constructed based on (i) the identifying elements, and (ii) the surrounding “auxiliary” terms that appear around the identifying elements. Next, the pattern set of C is expanded with new patterns which are created by joining existing patterns that share overlapping terms. Finally, each pattern is assigned a score showing its reliability as an annotation indicator for the particular GO concept it belongs to.

The GEANN annotation discovery phase takes the extracted patterns as its input, and produces a set of GO annotations for genes. For each pattern, GEANN looks for possible matches in Pubmed abstracts. The extracted patterns are *flexible* in that they match a class of phrases with close meanings. To this end, GEANN employs WordNet [3] to deduce *semantic closeness* of the words in the pattern matching process. WordNet is an online lexical reference system which organizes English nouns, verbs, adjectives and adverbs into synonym sets (synsets), each representing an underlying lexical concept. Given a pattern P and an excerpt T from a paper abstract, P matches T if (i) T includes the significant phrase S that constitutes the core of P, and (ii) there is at least one pair of words W_1 and W_2 that appears around S (in a certain *window size*) in P and T, respectively, such that W_1 and W_2 are semantically similar. As for the semantic similarity computation between words over WordNet, we employ edge distance-based and information content-based methods. More specifically, the edge-distance measure counts the number of relationships on the path from W_1 to W_2 in the WordNet hierarchy, while the information content-based measure relies upon the information content of the least common ancestor of W_1 and W_2 in WordNet as the similarity between W_1 and W_2.

Once a match for a pattern is detected, GEANN locates the genomic entity (if any) that is most likely to be associated by the matching phrase towards an annotation. Hence, similar to the training papers, genomic entities in each target paper are also tagged before the matching is finalized. Next, GEANN computes a match score which shows the likelihood that the matched phrase can be used as a supporting evidence for the new annotation. Finally, GEANN attempts to map the genomic entity located in an abstract to a gene or a set of genes in the database. If there exists such a mapping, the annotation is stored in the GEANN database to be included in the final discovered annotation set.

2. Methods and Algorithms

In this section, we present the algorithm sketches for the procedures that are described in the main manuscript.

2.1. Computing Significant Terms

Given (i) a GO concept C, (ii) the set Ann(C) of gene annotations by C, (iii) a database of paper abstracts (PubMed in our case), and (iv) a threshold value, the algorithm sketch in figure 2 computes the set of significant terms for C. For each term t that appears in an evidence paper of the input GO concept C, the algorithm simply computes the number of evidence papers (tf) that are associated with C, and that contain t, as well as the number of papers (idf) that contain t in the whole input paper database. Then, a simple *statistical enrichment score* is computed as (see the main manuscipt). The terms with enrichment scores below the input threshold are excluded

from the significant term list for C. Moreover, the terms that constitute the name of a GO concept that is being processed are by default considered to be significant terms.

```
Algorithm: ComputeSignificantTermSet
Input:  C:       Target GO Concept
        Ann(C):  Annotation Set of the target GO concept
        D:       A genomics paper database (e.g., PubMed)
        EThr:    Enrichment Score Threshold
Output: S(C):    The set of significant terms for the target GO concept
S(C)←{};
EP ← All evidence papers in Ann(C);
Terms ← All terms that appear in any paper in EP;
foreach(Term t in Terms) do
            idf← the number of papers in D, which contain t;
            tf← the number of papers in EP, which contain t;
            EScr←ComputeEnrichmentScore(tf, EP, idf, D);
            if(EScr ≥ EThr)then
                   S(C)←S(C)∪t;
end foreach
foreach(Term t in name(C)) do
      S(C)←S(C)∪t;
end foreach
return S(C);
```

Figure 2. Algorithm Sketch to Compute Significant terms

2.2. Computing Significant Phrases

Significant phrases are constructed out of significant terms through a procedure similar to the Apriori algorithm [24]. Given (i) a GO concept C, (ii) the set Ann(C) of gene annotations by C, (iii) a database of paper abstracts (PubMed in our case), (iv) a threshold value, and (v) the significant term set S(C) for C, the algorithm sketch in figure 3 computes the set of significant phrases for C. In the first phase, each pair of terms in S(C) are combined to create length-2 candidate phrases where "length" refers to the number of terms in a phrase. Then, the statistical enrichment score is computed for each candidate phrase, and those with enrichment scores below the input threshold are eliminated. Next, each pair of length-2 significant phrases is combined to obtain a length-3 candidate phrase, and similarly those with insufficient enrichment scores are eliminated. The procedure goes on in the same manner by creating length-(k+1) candidate phrases out of length-k phrases, and eliminating the ones with enrichment scores lower than the input threshold. In order for two significant phrases SP_i and SP_j of length-k to be *combinable* to produce a length-(k+1) candidate phrase, the last k-1 terms in SP_i should be the same as the first k-1 terms in SP_j. The procedure is repeated until no more new phrases can be created.

```
Algorithm: ComputeSignificantPhraseSet
Input:  C: Target GO Concept
        Ann(C): Annotation Set of the target GO concept
        D: A genomics paper database (e.g., PubMed)
        EThr:   Enrichment Score Threshold
        S(C):   The set of significant terms for the target GO concept
Output: SP(C):  The set of significant phrases for the target GO concept
SP(C)←{};
EP ← All evidence papers in Ann(C);
C_k←S(C);
C_k+1←{};
while(C_k is not empty) do
  foreach(Significant Term st_i in S(C)) do
```

```
      foreach(Significant Term st_j in S(C)) do
         if(st_i is_combinable_with st_j)then
            //string concatenation: st_i + " " + st_j
            candidateSP←Combine(st_i, st_j);
            EScr←ComputePhraseEnrichmentScore(candidateSP, EP, D);
            if(EScr ≥ EThr)then
               C_{k+1}←C_{k+1} ∪ candidateSP;
      end foreach
   end foreach
   SP(C)←C_{k+1} ∪ SP(C);
   C_k←C_{k+1};
end while
return SP(C);
```

Figure 3. Algorithm Sketch to Compute Significant Phrases

2.3. Constructing Patterns

Figure 4 below presents an algorithm sketch to construct regular patterns. Given (i) a GO concept C for which the patterns will be extracted, (ii) C's annotation set with corresponding evidence papers, (iii) Significant terms and phrases that are computed for C in the previous step, and (iv) a window size value, the algorithm returns the set of all regular patterns for C. More specifically, for each significant term or phrase TP, first, a pattern template is created, and its middle tuple is initialized to TP. Then, for each occurrence of TP in each evidence paper of C, a new pattern is created. And, the left and the right tuples of the new pattern are assigned to the surrounding words around TP in the currently processed evidence paper. Finally, the constructed patterns are returned as the output.

```
Algorithm: ConstructRegularPatterns
Input:  C: Target GO Concept
        Ann(C): Annotation Set of the target GO concept
        STP(C): Significant terms and phrases for the target GO concept
        WindowSize: The number of terms allowed in LEFT/RIGHT tuples

Output: RegPat(C): Regular patterns for the target GO concept

RegPat(C)←{};
EP ← All evidence papers in Ann(C);
foreach(Significant Term/Phrase tp in STP(C))do
   PatternTemplate temp←new PatternTemplate();
   temp.MiddleTuple←tp;
   foreach(Occurrence occ of tp in papers in EP) do
      Pattern newPattern←temp.CreatePatternInstance();
      newPattern.LeftTuple←occ.getLeftSurroundingWords(WindowSize);
      newPattern.RightTuple←occ.getRightSurroundingWords(WindowSize);
      RegPat(C)←RegPat(C)∪newPattern;
   end foreach
end foreach
return RegPat(C);
```

Figure 4. Algorithm Sketch to Compute Regular Patterns

Transitive Crosswalk

Figure 5 presents a simple algorithm sketch to construct side-joined patterns. Given a set of regular patterns, the algorithm simply checks each pair of regular patterns to see if the right tuple of the first pattern is the same as the left pattern of the second pattern. If this is the case, a new 5-

tuple side-joined pattern is created, and its tuples are initialized.

```
Algorithm: ConstructSideJoinedPatterns
Input: C: Target GO Concept
       RegPat(C): Regular patterns for the target GO concept

Output: SJPat(C): Side-joined patterns for the target GO concept
SJPat(C)←{};
foreach(Pattern pat1 in RegPat(C))do
    foreach(Pattern pat2 in RegPat(C))do
       if(pat1 = pat2)then
          continue;
       if(pat1.RightTuple = pat2.LeftTuple)then
          SideJoinedPattern sj←CreateEmptySideJoinedPattern();
          sj.LeftTuple1←pat1.LeftTuple;
          sj.MiddleTuple1←pat1.MiddleTuple;
          sj.RightTuple1←pat1.RightTuple;
          sj.MiddleTuple2←pat2.MiddleTuple;
          sj.RightTuple2←pat2.RightTuple;
          SJPat(C)←SJPat(C)∪sj;
    end foreach
 end foreach
 return SJPat(C);
```

Figure 5. Algorithm Sketch to Construct Side-joined Patterns

Middle Crosswalk

Figure 6 depicts an algorithm sketch to construct middle-joined patterns. For each pair (P1, P2) of patterns in the input pattern set, the algorithm checks for overlaps either between middle tuple of P1 and left tuple of P2, or between right tuple of P1 and middle tuple of P2. If any overlap is found, then, according to the cases which are enumerated in the main manuscript, a new 4-tuple middle-joined pattern is created.

```
Algorithm: ConstructMiddleJoinedPatterns
Input: C: Target GO Concept
           RegPat(C): Regular patterns for the target GO concept

Output: MJPat(C): Middle-joined patterns for the target GO concept
MJPat(C)←{};
foreach(Pattern pat1 in RegPat(C))do
    foreach(Pattern pat2 in RegPat(C))do
       if(pat1 = pat2)then
          continue;
       if(pat1.MiddleTuple ∩ pat2.LeftTuple ≠ ∅)then
          MiddleJoinedPattern mj←CreateEmptyMiddleJoinedPattern();
          mj.LeftTuple←pat1.LeftTuple;
          mj.RightTuple←pat2.RightTuple;
          mj.MiddleTuple1←pat1.MiddleTuple ∩ pat2.LeftTuple;
          if(pat1.RightTuple ∩ pat2.MiddleTuple ≠ ∅)then //Case (c)
             mj.MiddleTuple2←pat1.RightTuple ∩ pat2.MiddleTuple;
          else //Case (b)
             mj.MiddleTuple2←pat2.MiddleTuple
          else if(pat1.RightTuple ∩ pat2.MiddleTuple ≠ ∅)then //Case (a)
             MiddleJoinedPattern mj←CreateEmptyMiddleJoinedPattern();
             mj.LeftTuple←pat1.LeftTuple;
             mj.RightTuple←pat2.RightTuple;
```

```
            mj.MiddleTuple1←pat1.MiddleTuple;
            mj.MiddleTuple2←pat1.RightTuple ∩ pat2.MiddleTuple;
    end foreach
end foreach
return MJPat(C);
```

Figure 6. Algorithm Sketch to Construct Middle-joined Patterns

Pattern Matching

Figure 7 presents an overview of the algorithm for locating pattern matches. Given a pattern *Pat* to be searched in a set of papers *PaperSet*, and the GO concept that Pat belongs to, the algorithm returns a set of gene annotation predictions with their confidence scores. For each occurrence of Pat's middle tuple in a paper Pr in PaperSet, the corresponding left and right tuples are extracted from the surrounding words around the occurrence in Pr (lines 4-5). Then, Pat's left and right tuples are compared for semantic similarity to the left and right tuples that are just extracted from Pr. In the algorithm of figure 7, this comparison procedure is abstracted through the invocation of the function *ComputeMatchingScr (see the main manuscipt).*

```
Algorithm: SearchPatternMatching
Input: Pat: A Regular Pattern
PaperSet: A set of papers to be searched for Pat
C: The GO concept to which Pat belongs

Output: Ann: A set of annotations of the form (C, geneName, confidence)
1: Ann←{};
2: foreach(Paper pap in PaperSet)do
3:   foreach(Occurrence occ of pat.MiddleTuple in pap)do
4:     tempLeftTuple←occ.getLeftSurroundingWords(pat.LeftTuple.Length);
5:     tempRightTuple←occ.getRightSurroundingWords(pat.RightTuple.Length);
6:     leftTupleMatchScr←ComputeMatchingScr(pat.LeftTuple, tempLeftTuple);
7:     rightTupleMatchScr←ComputeMatchingScr(pat.RightTuple, tempRightTuple);
8:     matchScore←(leftTupleMatchScr + rightTupleMatchScr)/2;
9:     if(matchScore > 0)then
10:       confidence←pat.GetScore() * matchScore;
11:       geneName←LocateGeneAroundMatch(occ, pap);
12:       Ann←Ann ∪ (C, geneName, confidence);
13:    end foreach
14: end foreach
15: return Ann;
```

Figure 7. Algorithm Sketch for Pattern Matching

2.7. Pattern Matching

In figure 8, we present a sketch of the implementation for *ComputeMatchingScr* function which is called by the main algorithm in figure 7. In order to compute the overall semantic similarity between sets of words based on the similarities between individual word pairs, we utilize an open source software library [32] which uses the Hungarian method [16] to solve the problem as follows. Given two word sets, WS1 and WS2, let n be the number of words in WS1, and m be the number of words in WS2 (lines 1-2). First, a semantic similarity matrix, R[n, m], containing each pair of words in WS1 and WS2 is built (line 3), where R[i, j] is the semantic similarity between the word at position i of WS1 and the word at position j of phrase WS2, which can be computed using either of the measures explained above (line 8). Thus, R[i, j] is also the weight

of the edge from i to j. The problem of computing the semantic similarity between two sets of words WS1 and WS2 is considered as the problem of computing the maximum total matching weight of a bipartite graph [16]. This makes sense since WS1 and WS2 are disjoint in the sense that the comparisons are always made between word pairs that belong to different sets. Finally, the Hungarian method [16] is used to solve problem of computing the maximum total matching weight of a bipartite graph.

```
Algorithm: ComputeMatchingScr
Input: WordSet1: A set (bag) of words
       WordSet2: Another set (bag) of words to be compared to WordSet1

Output: SScr: Semantic Similarity Score between WordSet1 and WordSet2
n←WordSet1.Size();
m←WordSet2.Size();
R[n,m]←Matrix[n,m]; // Initialize semantic similarity matrix
for(i←0;i<n;i++)do
   word1←WordSet1[i];
   for(j←0;i<n;i++)do
     word2←WordSet2[j];
     //Compute semantic similarity between word1 and word2 using either
//edge distance- or information content-based measure
     R[i,j]←ComputeSemanticSimilarity(word1,word2)
   end for
end for
//Construct bipartite graph where nodes corresponds to the words in
//WordSet1 and WordSet2, and the weights are assigned from R
BipartiteGraph←ConstructBipartiteGraph(WordSet1, WordSet2, R);
//Compute the maximum total matching weight using Hungarian Method
maxMatchingWeight←HungarianMaxTotalMatchWeight(BipartiteGraph);
return maxMatchingWeight;
```

Figure 8. Algorithm Sketch for Semantic Similarity Computation between Sets of Words

3. Additional Results and Discussion

3.1. Overall Performance and Named Entity Tagger Errors

The accuracy of the tagging gene/gene products in the text influences the association of a pattern to a genomic entity. However, named entity taggers (NETs) are still not perfect in locating all the gene names correctly in textual data. As an example, the NET Abner that is used by GEANN has 68% precision at 77% recall [33]. Hence, the results given in observation 1 (in the main manuscript) also involve erroneous cases resulting from the NET. Therefore, for an accurate evaluation of GEANN system alone, one needs to quantify the cases where low recall values are due to the imperfections of the tagger, and not due to the deficiencies of GEANN. It may be quite hard to exactly quantify those error cases where the tagger prevents GEANN from predicting an annotation accurately. Therefore, we take a minimalist approach, and attempt to quantify the portion of the errors that is guaranteed to be due to the fact that the NET has missed some of the genes. The heuristic that we employ is as follows.

H1 *(Tagger-missed genes):* If none of the synonyms of a gene has been recognized by the tagger in any of the papers which are associated with the GO concept G, then label the gene as a *tagger-missed gene*, and remove it from the paper set associated with G.

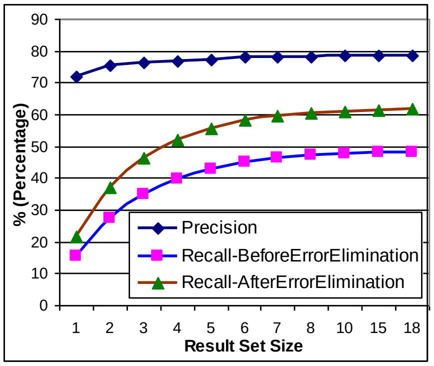

Figure 9. Overall System Performance & Approximate Effect of the Error due to the Named Entity Tagger

Thus, from the middle line of figure 9, we have

Observation: *After eliminating tagger-missed genes, the average recall of GEANN has increased to 61% from its previous value 48% at the precision level of 78%.*

Clearly, heuristic 1 underestimates the actual error rate of the named entity tagger. More specifically, in contrast to the implicit assumption of the heuristic, recognizing a gene in at least one of the papers may not guarantee that the gene is located at the text position it is expected. It is crucial to be able to recognize the gene in the papers that contain texts matching at least one pattern rather than in an unrelated paper, that is, the heuristic used to model the tagger error rate assumes the minimal error rate, but the actual error rate may be much higher. In addition, eliminating tagger-missed genes will not improve the precision as the elimination process only narrows down the gene set that is missed, and has no effect on the discovered gene set. Therefore, precision is plotted only once in figure 9. For the experiments, we consider the negative effect due to NET errors.

3.2. Annotation Accuracy across Different Subontologies in GO

In this section, evaluate the accuracy of GEANN across the three different subontologies of GO, namely, biological process, molecular function, and cellular location. To this end, the GO concepts are grouped according to the subontologies they belong to. Then, the same steps described in experiment 1 are run once again, but, this time, average values are computed within the individual groups rather than out of the whole concept set used for the evaluation. Figure 10 plots the precision/recall values of the three different subontologies of GO (MF: Molecular function, BP: Biological Process and CC: Cellular Component).

Observation 3: *In terms of precision, GEANN provides the best precision for the concepts from cellular component (CC) and molecular function subontologies where precision is computed as 80% while biological process (BP) subontology yields the highest recall (63% at 77% precision).*

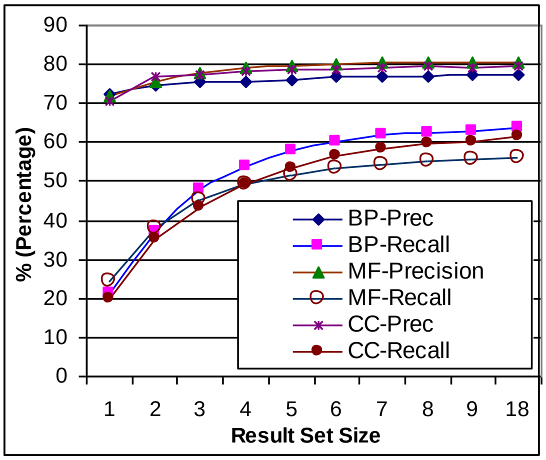

Figure 10. Annotation accuracy across different subontologies in GO

The fact that MF subontology provides better precision may be due to the fact that functionality concepts in GO are more specific in the MF subontology than biological process names. This is mainly because biological process concepts refer to biological pathways, and pathways are more general biological abstractions in comparison to the specific functionalities of enzyme proteins/genes, a number of which is included in each pathway. As for the CC subontology, higher precision values may be because, in contrast with other subontologies, the variety of the words to describe the cellular location is perhaps much lower, and the fact that the CC ontology is the smallest among the three GO subontologies supports our reasoning.

4. Enhancing Recall

As illustrated through the experimental results in the main manuscript and the supplementary material document, an inherent drawback of pattern-based text mining systems is the fact that their recall performance is frequently low. In this section, we describe and evaluate two different approaches to obtain annotation predictions with high recall: (i) through a probabilistic annotation framework, and (ii) by adjusting statistical enrichment threshold value.

4.1. A Probabilistic Annotation Framework

GEANN models the surrounding words around annotated genes in training paper set as patterns. In order to take advantage of relatively higher precision due to the use of structural patterns and to alleviate the strictness of pattern-based systems, in this section, we model the surrounding text around target gene names in terms of multiple *ordered pairs* of words. Then, based on the frequency of each ordered pair in the training data, we compute the likelihood that a given ordered pair appears in the neighborhood of a gene which is annotated with a certain GO concept. In other words, we model the neighborhood of an annotated gene as a set of ordered pairs of words associated with probability scores.

Definition *(k-neighborhood of a gene): Given a textual excerpt T from a paper P where stopwords are eliminated from T, and a gene g that appears in T at position i, let T(j) represent the word that appears at position j in T. Then, the k-neighborhood k-Nhood(g, T) of g is an*

ordered list of words which are located at most k-words apart from g in T, that is, k-Nhood(g, T) = (T(n) | i-k ≤ n ≤ i+k and T(i)=g and n≠i). And, k is called the radius of the neighborhood.

Example 5: Consider the excerpt T from the abstract of a paper [39] which describes the role of gene *Kap104* in cell cycle.

T = " … **implies** a **novel role** for *Kap104* in **cell cycle progression** …"

3-NHood(*Kap104*, T) = (imply, novel, role, cell, cycle, progress) and 2-Nhood(*Kap104*, T) = (novel, role, cell, cycle) after stopwords are eliminated and the remaining words are represented in their base forms.

Next, in order to represent the structure in the neighborhood of a gene in a flexible manner, we construct ordered pairs of words from the k-neighborhood of a gene. We give an example.

Example 6: For the gene *Kap104* in the previous example, assume that the neighborhood radius is 3. Then, the ordered term pair representation of 3-NHood(*Kap104,* T) is as follows.

$$\text{3-NHood(Kap104, T)} = \left\{ \begin{array}{lllll} \text{(imply, novel)} & \text{(novel, role)} & \text{(role, cell)} & \text{(cell, cycle)} & \text{(cycle, progress)} \\ \text{(imply, role)} & \text{(novel, cell)} & \text{(role, cycle)} & \text{(cell, progress)} & \\ \text{(imply, cell)} & \text{(novel, cycle)} & \text{(role, progress)} & & \\ \text{(imply, cycle)} & \text{(novel, progress)} & & & \\ \text{(imply, progress)} & & & & \end{array} \right\}$$

Figure 11. Ordered Term Pair Representation for *Kap104*

Definition *(Ordered-pair representation of a gene's k-neighborhood): Given a textual excerpt T from a paper P, a gene g, and a k-Nhood(g, T) of g in T, let* w_i *denote the word at position i in k-NHood(g, T). Ordered pair representation for k-NHood(g, T) is k-OPR(g, T) = {(* w_i*,* w_j*) | i < j}.*

Remark: *The number of ordered pairs in k-OPR(g, T) of a gene g is* $\frac{(k-1)(k-2)}{2}$.

After creating ordered pair representation for each appearance of genes in the training paper set, we compute a probability score for each ordered-word-pair *(*w_i*,* w_j*)* based on the frequency of *(*w_i*,* w_j*)* in all possible neighborhoods.

Definition *(Probability of an ordered-word-pair): Given a set S of k-OPR(g,* T_i*)s where g is a gene in the training data set, and an ordered-word-pair (*w_i*,* w_j*), let S' be subset of S such that ∀ k-OPR(g,* T_i*) ∈ S', (*w_i*,* w_j*) ∈ k-OPR(g,* T_i*). Then, the occurrence probability P((*w_i*,* w_j*)) of (*w_i*,* w_j*) is P((*w_i*,* w_j*)) =|S'| / |S|. Note that P((*w_i*,* w_j*)) ≠ P((*w_i*,* w_j*))*

Example 7: Consider the textual excerpts T_1 [39] and T_2 [40], which contain neighborhoods (with radius 3) for genes *Kap104* and *Cdc6*, respectively, as shown in figures 11 and 12.

T_1 = " … **implies** a **novel role** for *Kap104* in **cell cycle progression** …"

T_2 = "… **cells degrade ubiquitinated** *Cdc6* every **cell cycle** at the **beginning** …"

Then, the set S of all neighborhood sets S = {*3-NHood(Kap104,* T_1*), 3-NHood(Cdc6,* T_2*)*}, and occurrence probability of ordered-pair *(cell, cycle)* is P((*cell, cycle*)) = 1 as it appears in all 3-neighborhoods in S, while P((*cycle, progress*)) = 0.5 since it appears only in *3-NHood(Kap104,* T_1*)*.

3-NHood(Cdc6, T_2) =	*(cell, degrade)*	*(degrade, ubiquitin)*	*(ubiquitin, cell)*	~~*(cell, cycle)*~~	*(cycle,begin)*
	(cell, ubiquitin)	*(degrade, cell)*	*(ubiquitin, cycle)*	~~*(cell, begin)*~~	
	(cell, cell)	*(degrade, cycle)*	*(ubiquitin, begin)*		
	(cell, cycle)	*(degrade, begin)*			
	(cell, begin)				

Figure 12. Ordered Term Pair Representation for *Cdc6*

Note that since the word “cell” appears two times in the neighborhood, the duplicate ordered pairs (shown with a strikethrough line) are eliminated.

The probability of an ordered-pair is dependent upon the size of the reference gene neighborhood set S according to which it is defined. When S equals the set of all gene neighborhoods in evidence papers of a GO concept G, the probability of an ordered-pair (w_i, w_j) is denoted as $P_G((w_i, w_j), G)$. Moreover, when S equals the set of all gene neighborhoods in evidence papers that are associated with any GO concept in a set D of GO concepts, the probability of an ordered-pair (w_i, w_j) is denoted as $P_D((w_i, w_j), D)$.

Next, based on the probability of an ordered-pair both in the context of a GO concept and in the context of a database D of GO concepts, we define the *enrichment ratio* of an ordered pair as a measure of how related an ordered pair is to a GO term.

Definition *(Enrichment Ratio of an Ordered-Pair): Given an ordered pair (w_i, w_j), a database D of GO annotations, and a set D’ of annotations for a particular GO concept G such that $D' \subseteq D$, the enrichment ratio of (w_i, w_j) with respect to G and D is $E((w_i, w_j), G, D) = P_G((w_i, w_j), G)/ P_D((w_i, w_j), D)$.*

Finally, we compute the score of candidate annotation of a gene *g* with a GO concept G by summing the enrichment ratio of each ordered pair in its *g*’s neighborhood.

Definition *(Score of a candidate annotation): Given a GO concept G, a database D of GO concepts, a neighborhood radius k, a textual excerpt T from a paper, and the ordered-pair representation k-OPR(g, T) for a k-neighborhood k-NHood(g, T) of a gene g in T, the score S(g, G, D) of candidate annotation of g by G in k-NHood(g, T) is*

$$\Sigma_{(wi, wj) \in k\text{-}OPR(g, T)} E((w_i, w_j), G, D)$$

If a gene has multiple neighborhoods (i.e., multiple occurrences/mentions), then the score of a candidate annotation for a gene is the sum of all its neighborhoods as each neighborhood is considered as a distinct evidence for the annotation.

Precision/Recall Analysis:

In order to measure the accuracy of the approach described in this section, we perform k-fold cross validation, and we employ the same assumptions which are also presented in the results section of the main manuscript. Figure 13 presents the precision and recall values at different top-k values where the neighborhood radius is set to 10. Since the results set sizes for GEANN and the probabilistic approach are different, in this section, we do not show their performance results on the same graph. For GEANN’s performance results, please see section 5.

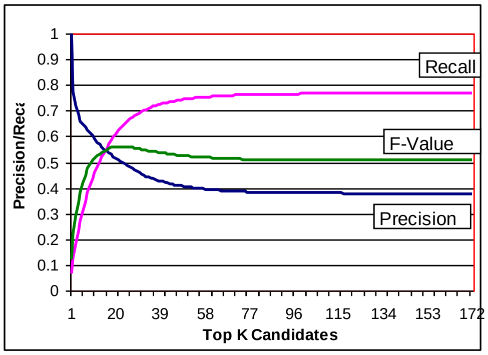

Figure 13. Performance of the Probabilistic Approach

Observation 13: *The probabilistic approach provides the maximum F-value of 0.56 when precision is 51% and recall is 62%, while pattern-based GEANN provides the maximum F-value of 0.68.*

Observation 14: *At recall of 61% which is the maximum recall that GEANN can achieve at its maximum precision level, the probabilistic approach has a precision of 51% while GEANN has precision of 78%.*

Observation 15: *The probabilistic approach can reach to higher recall values (77% at the maximum) which is significantly higher than what GEANN provides (61% at the maximum).*

Since pattern-based GEANN algorithm is much more stricter than the probabilistic approach, GEANN achieves higher precision at the cost of missing some of the annotations which leads to low recall values. The bottom-line lesson that one can derive from the above observations is that, for application settings that require high precision, the pattern-based GEANN is the option to be employed, while, for some other application settings that put more value on completeness (hence high recalls), the probabilistic approach can be preferred over GEANN. One such setting that may emphasize high recalls is when there exist a large number of curators available, who can further filter the false positives returned by the probabilistic approach, and have a more complete set of annotations. On the other hand, for settings with minimal or no curation facility (e.g., an unsupervised web-based annotation tool), GEANN may be more useful with lower number of *false positives*.

Assessing Different Choices on Neighborhood Radius

The probabilistic approach constructs gene neighborhoods (hence ordered-pair sets) based on the radius of the neighborhood variable. In this section, we evaluate the effect of different radius choices on the overall performance of the probabilistic approach. We compare the results based on (a) the average precision and recall that are computed over different top-k values by varying k, and (b) the maximum precision/recall that can be obtained. The table below summarizes the results for different values of the neighborhood radius.

Radius	Average Precision	Average Recall	Precision at Max Recall	Max Recall
1	0.61	0.39	0.59	0.40
2	0.50	0.62	0.46	0.68
3	0.44	0.68	0.41	0.74

7	0.42	0.71	0.38	0.77
10	0.42	0.71	0.38	0.77
14	0.42	0.71	0.38	0.77

Table 6.1 The performance of the probabilistic approach at different neighborhood radius values

Observation 16: *Smaller neighborhood radius values provide higher precision but lower recall values, while larger neighborhood radius values lead to higher recall, but lower precision values.*

As the neighborhood size gets larger, the ordered-pair sets that are taken into consideration during the enrichment score analysis are constructed out of the words that are far from the candidate gene that ocurs in the text. Since, words that are far from a gene occurrence most of the time are not directly related to or refer to the candidate gene, the reliability of such words as a basis for a possible annotation decreases. Hence, the overall precision decreases as the neighborhood sizes get larger. As for low recall values at smaller neighborhood radius values, the only way a candidate gene can get a non-zero score so that it could be considered for the final result set is that it should have at least one ordered-pair *(Wi, Wj)* in its neighborhood such that *(Wi, Wj)* also appears in one of genes' neighborhoods in the training data. As we presented through a remark above, the number of ordered pairs in k-neighborhoods of a gene G is $O(k^2)$) where k is the neighborhood radius. As the size of the ordered-pair representation of a gene's k-neighborhood decreases due to the smaller radius selection, the probability that its neighborhood would have a common ordered pair with any of the genes in the training data decreases. Hence, the number of candidate genes which are not considered in the final result set increases, which leads to missing larger number of genes compared to the cases where the neighborhood radius is set to larger values.

Incorporating Constraints for Ordered-Pairs

Presently, the probabilistic approach utilizes all ordered pairs for the candidate gene set generation, regardless of the enrichment values of the ordered-pairs that are extracted from the training data. In this section, we study enforcing a threshold constraint on the enrichment scores of ordered-pairs so that only those ordered-pairs with enrichment scores over a given threshold are utilized to locate and score candidate test genes. To this end, similar to the evaluation scheme of section 6.1.2, we present tabular results for average and max values of precision and recall at different enrichment ratio thresholds. Table 6.2 presents the results of this experiment where the neighborhood radius is set as 7.

Enrichment Ratio Threshold	Average Precision	Average Recall	Precision at Max Recall	Max Recall
0	0.42	0.71	0.38	0.77
0.1	0.59	0.39	0.57	0.43
0.2	0.62	0.36	0.58	0.42
0.3	0.69	0.41	0.66	0.48

Table 6.2 The performance of the probabilistic approach at different enrichment ratio thresholds

Observation 17: *Incorporation of the enrichment ratio threshold increases the precision as much as 60% while the recall decreases at almost the same rate.*

Since ordered-pairs with high enrichment ratios are expected to be more related to a particular GO term, it is expected that the precision will increase. Due to the same reasoning that is presented in section 5.2 to explain the low recall values for smaller neighborhood radius sizes, depending on the decrease in the total number of ordered data sets, the number of candidate genes with no overlapping ordered-pairs with the training set of genes increases, which leads to lower recall values at high enrichment thresholds.

Once again, depending on the application type, one may choose to apply lower or higher enrichment ratio thresholds to adjust the accuracy of the probabilistic approach.

4.2. Adjusting Statistical Enrichment Threshold

In this experiment, our goal is to study how the accuracy, particularly recall, of GEANN changes at different statistical enrichment (SE) thresholds, which is used to eliminate words that are not useful for the GO concept being annotated. Figure 14 shows the precision and the recall at different SE threshold values.

Observation 18: *The maximum recall of 87% is obtained when the enrichment threshold is 2 where F-value also reaches to its maximum value of 0.76.*

Observation 19: *Adjusting enrichment threshold to lower values results in higher recall than the maximum recall value provided by the probabilistic approach of section 6.1.*

Observation 20: *As the enrichment threshold increases, GEANN's precision increases while the recall and F-values decrease. The fact that F-value also decreases by increasing the enrichment threshold indicates that recall decreases more than the increase in precision.*

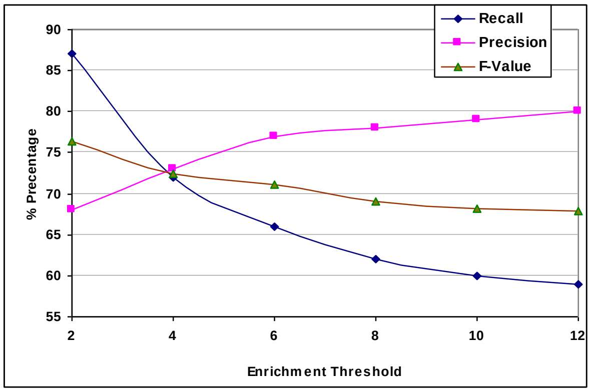

Figure 14. Precision/Recall at Different Enrichment Thresholds

It is expected that, with increasing enrichment threshold values, the precision should increase since, as the enrichment threshold increases, more significant terms are eliminated from the initially selected set of terms. Although the F-value is the highest at low enrichment thresholds, it does not directly imply that lower enrichment thresholds should be preferred because, at lower thresholds, the generated significant term list may contain irrelevant terms, which are not intuitively descriptive for the corresponding GO concept. Moreover, since the targeted databases are quite large (PubMed with 15 million papers, for instance), increasing the precision is more crucial than getting higher recalls in the context of our problem. Hence, the enrichment filter provides a controlled mechanism to achieve higher precision in large text databases. Therefore,

at the expense of losing some F-value, throughout the above experiments presented in previous sections, we set the enrichment threshold at a moderate value of 6. However, for the comparative study in section 5.4, the enrichment threshold is set to 2 since only F-values are known for the other studies, and GEANN provides the highest F-values at lower enrichment thresholds.

Observation 21: *At higher enrichment thresholds, no significant terms satisfying the enrichment threshold could be found for several GO concepts. At the enrichment threshold of 10, two GO concepts, and at the enrichment threshold of 12, five GO concepts were removed from the experimental set as no significant terms (hence no patterns) could be generated for these GO concepts.*

Observation 22: *All GO concepts for which no significant term could be found at higher enrichment thresholds reside at GO levels 5 or lower.*

The above observation indicates that high enrichment thresholds mostly hurt general GO concepts at lower levels (5 or less) of GO (where the root resides at level 1), which makes sense since the concepts at higher levels are quite diverse in that they span a number of distinct genomic or biological titles in a single context. Therefore, it is less likely or more difficult to find terms that can only be attributed to such concepts, but not to the others.

5. Related Work

Raychaudhuri et al. [5] and Izumitani et al. [6] employ machine learning techniques for gene annotation via classification of articles that are associated with each gene into GO concepts. As discussed in the previous section, although GEANN is more flexible in terms of its assumptions and granularity of the resulting annotations, its performance is still comparable to these systems. Asako et al. [12] employs actor-object relationships by analyzing the sentences syntactically from the NLP (Natural Language Processing) perspective. This system is optimized for the biological process subontology, and the actor-object relationship may not always apply to other ontolologies, e.g., cellular location. Finally, the system is semi-automatic, i.e., it requires human input, and manually created patterns and regular expressions. The system is reported to achieve 36% precision at 51% recall.

Ravichandran and Hovy [14] studied surface text patterns for open-domain question answering systems. For a birthday question (e.g., “When was X born?”), for instance, among the patterns extracted by the system are “<NAME> was born on <ANSWER>”, “born in <ANSWER>, NAME”. One of the shortcomings of this approach is that some of the extracted patterns are very general (e.g., <ANSWER>, <NAME> extracted for “what is x?” type of question). Hence, such patterns match to many false positives. Besides, the extracted patterns cannot locate long distance dependencies which involve a set of words between the answer and the question term in a sentence.

Mann [15] explores techniques to automatically construct a fine-grained proper noun ontology using textual co-occurrence patterns. To this end, the patterns are sequences of part-of-speech tags. The presented approach in this work only considers the syntactical structure of phrases in terms of their part-of-speech tags. Hence, Mann ignores semantic features conveyed in individual terms of a pattern. Another limitation of this technique is that the user has to provide part-of-speech tag sequences to the system.

Fleischman et al. [41] proposes extensions to Mann’s work. The extension involves the usage of

appositions as well as noun/proper noun constructions, and the incorporation of filtering mechanisms through the employment of classifiers (e.g., Naive Bayes, SVM) in order to eliminate incorrect pattern matches. Although this methodology works for the extraction of concept instance relationships, it does not eliminate the inherited limitations of the framework [15] that it extends. In addition, during the filtering stage, in order to train the classifiers, Fleischman et al. creates a hand-tagged set of 5,000 pattern output labeled as legitimate or illegitimate. In the case of Pubmed, preparing a hand- tagged set from 15 million Pubmed abstracts would not be a practical approach.

In another study, Fleischman and Hovy [17] present a supervised learning method to automatically classify person instances in eight fine-grained subcategories. This work is very similar to our flexible pattern approach in that it uses WordNet to deduce semantic closeness of words/phrases, and takes into consideration word frequency (topic signatures [17]) similar to what we call the significant word model. However, the proposed method relies on a large training set which is not available for our case as the number of referred Pubmed evidence articles for each GO concept is not large. Besides, we use significant terms to construct additional patterns so that we can locate additional semantic structures as indicators of annotation evidence while this article only considers the target instance as the base of its patterns. Last, but not the least, the framework proposed in this article only considers semantic closeness of frequent words, whereas we also take into account the semantic closeness of the surrounding terms of a pattern (i.e., terms in left and right tuples of a pattern).

Riloff [7] proposes a technique to extract the patterns from a large text corpus. His technique relies on some pre-defined 15 heuristic rules. The technique is also completely mechanical, and ignores the semantic side of the patterns. In addition, patterns are strict in that they require word-by-word exact matching.

Brin's DIPRE [8] is one of the pioneering systems in the information extraction field in recent decades. This work is motivated by extracting instances of a given relation (e.g., relation of books (author, title)) from web documents. DIPRE needs an initial set of seed elements as input, and uses the seed set to extract the patterns by analyzing the occurrences of seed instances in web documents. The algorithm in its next step uses newly discovered instances to generate new patterns.

SNOWBALL [9] extends DIPRE's pattern extraction system by introducing the use of named-entity tags, and pattern reliability evaluation techniques. Rather than assigning a fixed set of terms as the prefix and the suffix of the patterns, SNOWBALL employs a probabilistical approach. In this context, it attempts to accommodate all of the possible terms that have been seen in the previous occurrences of the pattern under varying scores according to their appearance frequencies. Later, the occurrence frequencies are used during the evaluation of matches to the patterns to omit false matches. Although SNOWBALL introduces a frequency-based pattern evaluation model, both SNOWBALL and DIPRE impose strict pattern structures that require exact keyword matches. Hence, if a prefix or a suffix has not been seen before, it has no chance of matching patterns ignoring the fact that it may well have a close semantic distance to the pattern prefix/suffix under consideration. In addition, these studies differentiate from our approach in that they only consider the relation instances to construct their patterns, and do not deal with other possible significant terms that may indicate a relationship being sought.

Etzioni et al. [10] developed a web information extraction system called KnowItAll. KnowItAll

intends to automate the discovery of large collection of facts in web pages. Since its working domain is www, the framework assumes information redundancy. Hence, it is developed with the postulation that creating patterns that match only simple phrases containing facts would be enough to achieve high precision and recall in the result set. To this end, they propose generic patterns which are instantiated by concepts and relationships of a given ontology. Provided with such an ontology and pattern instances, the system poses queries to different search engines with some keywords representing a pattern, and extracts the instances of a concept or relationship in the ontology. Despite the fact that the proposed framework scales well for web documents, this approach does not seem applicable to the mining of Pubmed abstracts for GO annotation evidences. This is because (i) the assumption of information redundancy does not hold for PubMed abstracts as each article is a peer-reviewed study, and contains original knowledge which is not repeated most of the time, (ii) dealing with only simple phrases and ignoring complicated sentences may decrease the precision and recall of our mining system as most of the sentences in scientific abstracts are not as simple as those accommodated on the web, and (iii) KnowItAll assumes that pattern templates are given by the user; however, our approach is to learn these templates from the existing annotations so that we can make use of the effort that has already been spent to manually curate the annotations.

Recently, in order to evaluate the existing annotation systems, the BioCreative contest [13] has been held. The second task of this contest is extracting the annotation phrases when given an article and a protein. The results [11] submitted by each participant were evaluated manually by the experts, and most of the evaluated systems had low precision (46% best performing system) with no recall calculation.

6. Future Work

GEANN can be extended in the future by addressing the following issues.

Data Reconciliation: In this study, reconciliation of the genomic entity set extracted from the text with genes and proteins of a well-known data source (e.g., GenBank), missing in other related work, is one of the significant steps within the annotation procedure. GEANN utilizes the synonym information stored in GenBank to identify the discovered entities within the gene and protein set of GenBank. Genomic entities may have several synonyms for historical purposes, and as their functionality is better characterized through wet-lab experiments, new names are assigned to describe their newly discovered functions. Usually, the old names are also kept for back-compatibility purposes. Obviously, synonym information may not be enough to uniquely characterize a gene in a large biological data source. As a future extension to GEANN, definitions and synonyms from variety of other data sources can be merged into a single extended genomic entity definition by taking advantage of external links pointing to the same entity in different data sources. Then, the extended definition can be attempted to match the surrounding words around the tagged gene/protein in the text, and the best match can be chosen.

Utilizing Concept Hierarchies: GEANN can be improved by exploiting the hierarchical structure that the GO concepts are organized. Currently, GEANN utilizes GO mostly as a data source, and does not take advantage of the internal organization among the concepts. The only structural property it exploits is during the accuracy analysis of predicted annotations, where an annotation to the parent of concept T is considered to be correct if the genomic entity is already annotated with concept T. This is also known as *true-path rule*. This rule can also be utilized during the

creation of extraction patterns, and pattern matching stages. For instance, the pattern set for the concept T can be enriched by including the patterns created for the descendants of concept T in the GO hierarchy. In addition, extraction patterns may be shared among the concepts which are not on the path. Through shared patterns, one can actually walk across the concepts in GO and utilize the additional patterns that would be obtained from the destination concept's prediction list. In this way, concepts that do not have many extraction patterns may have better recall values. This may lead to many false positives; hence, one needs to find measures to evaluate the strength of the predictions obtained through the GO concept walk.

Evidence Type Inference: Each annotation is given an evidence code specifying the way annotation has been established. GEANN currently ignores evidence codes and considers them of single type without any differentiation. As a GEANN extension, after the prediction stage, one more step can be applied to infer an evidence code for the newly discovered annotation. To this end, new extraction patterns can be constructed based on the existing annotation information. However, the evidence type is usually fairly implicit within the text; so extracting such information may require different pattern extraction techniques. One direction to proceed in this context is to consider the whole abstract as a single entity, and create model(s) of evidence code that will fit to the whole abstract, not just a single expression or sentence. For this purpose, sequence of indicator terms can be computed with their relative position information in account so that an evidence code skeleton for the whole abstract can be created. During the matching process, the existence of identifying terms and their relative positions will define the degree of conformity for the evidence code under consideration.

Entity Tagging: As GEANN depends on an external named entity tagger to recognize the gene/protein names in the text, it inherits the errors caused by the employed tagger. In this study, we developed a simple heuristic to approximate the effects of the error in named entity detection on GEANN's performance. The assumption that if a gene/protein is recognized in at least one sentence of an abstract, then it must have been recognized in all of the other articles does not completely characterize the error rate due to the named entity tagger. This is mainly because named entity taggers are more than syntactical keyword recognizers as they also take into account the surrounding contextual terms to disambiguate between real genomic entity names and homonym words used with different meanings. Hence, the assumption does not cover all the erroneous cases of the tagger, and, in practice, the contribution of the tagger error to performance decrease may be much larger. Exploring a better way of characterizing the tagger errors would be useful in order to have a better idea of the overall system performance. Obviously, the approach we took is a bottom-line approach which guarantees a lower bound for the tagger error rate.

Annotation Time: In this paper, our primary focus is on the accuracy of the algorithm. Hence, we have not made a special effort on the time efficiency of the system. Nevertheless, we have measured the annotation time for 1,000 abstracts, which is about 378.3 seconds for a GO concept. This can be further improved with additional indexing and in-memory processing of some of the statistical calculations if the database is small enough. As an example, the similarity between the words in WordNet can be pre-computed and stored in a database or in main memory to avoid similarity computations during the annotation.
