# Supplementary material for: Discovering gene annotations in biomedical text databases
Source: BMC Bioinformatics. 2008 Mar 6;9:143. doi: 10.1186/1471-2105-9-143 (PMC2335285; doi:10.1186/1471-2105-9-143)
Supplement: Additional file 2 — Appendix 1. This file contains the experimental GO concept set along with overall precision/recall values for each GO concept. [file 1471-2105-9-143-S2.pdf]

Appendix 1 – Experimental GO Concept Set

GOID	*Precision*	*Recall*	*GO Level*	*Subontology*	*Description*
GO:0000074	51.7	76.7	6	biological_process	regulation of progression through cell cycle
GO:0000122	69.6	50.2	10	biological_process	negative regulation of transcription from RNA polymerase II promoter
GO:0000398	80.6	88.0	11	biological_process	nuclear mRNA splicing via spliceosome
GO:0001501	80.1	89.7	5	biological_process	skeletal development
GO:0003702	38.8	77.7	3	molecular_function	RNA polymerase II transcription factor activity
GO:0003704	68.1	64.4	4	molecular_function	specific RNA polymerase II transcription factor activity
GO:0003713	26.6	58.9	6	molecular_function	transcription coactivator activity
GO:0003714	71.5	57.9	6	molecular_function	transcription corepressor activity
GO:0003723	26.1	64.6	4	molecular_function	RNA binding
GO:0003924	49.8	77.3	8	molecular_function	GTPase activity
GO:0004672	35.6	89.3	6	molecular_function	protein kinase activity
GO:0004674	40.8	74.6	7	molecular_function	protein serine threonine kinase activity
GO:0004725	64.6	90.6	8	molecular_function	protein tyrosine phosphatase activity
GO:0004842	79.2	62.7	7	molecular_function	ubiquitin protein ligase activity
GO:0004871	29.2	75.0	2	molecular_function	signal transducer activity
GO:0004888	63.1	95.2	4	molecular_function	transmembrane receptor activity
GO:0005102	66.7	100.0	4	molecular_function	receptor binding
GO:0005198	33.3	100.0	2	molecular_function	structural molecule activity
GO:0005200	75.5	81.3	3	molecular_function	structural constituent of cytoskeleton
GO:0005215	35.5	95.2	2	molecular_function	transporter activity
GO:0005489	52.0	90.5	1	molecular_function	electron transporter activity
GO:0005509	43.8	90.6	5	molecular_function	calcium ion binding
GO:0005524	61.0	86.7	6	molecular_function	ATP binding
GO:0005554	66.7	100.0	2	molecular_function	molecular function unknown
GO:0005576	43.9	100.0	2	cellular_component	extracellular region
GO:0005578	68.0	96.4	4	cellular_component	extracellular matrix (sensu Metazoa)
GO:0005615	39.9	74.6	4	cellular_component	extracellular space
GO:0005624	15.7	91.7	5	cellular_component	membrane fraction
GO:0005625	18.1	83.3	5	cellular_component	soluble fraction
GO:0005635	60.2	71.3	10	cellular_component	nuclear envelope
GO:0005643	83.8	71.8	13	cellular_component	nuclear pore
GO:0005654	42.6	70.4	11	cellular_component	nucleoplasm
GO:0005730	73.3	71.7	11	cellular_component	nucleolus
GO:0005741	71.7	82.7	12	cellular_component	mitochondrial outer membrane
GO:0005743	80.1	86.1	12	cellular_component	mitochondrial inner membrane
GO:0005759	82.2	77.5	11	cellular_component	mitochondrial matrix
GO:0005768	81.7	78.0	8	cellular_component	endosome
GO:0005777	82.5	78.5	9	cellular_component	peroxisome
GO:0005783	44.5	61.7	8	cellular_component	endoplasmic reticulum
GO:0005789	62.0	72.2	10	cellular_component	endoplasmic reticulum membrane
GO:0005794	68.8	74.0	8	cellular_component	Golgi apparatus
GO:0005829	46.0	73.3	8	cellular_component	cytosol
GO:0005856	62.7	95.2	8	cellular_component	cytoskeleton
GO:0005935	73.7	62.1	5	cellular_component	bud neck
GO:0005975	75.0	100.0	5	biological_process	carbohydrate metabolism

GO:0006260	71.4	94.3	7	biological_process	DNA replication
GO:0006281	75.8	82.9	7	biological_process	DNA repair
GO:0006350	48.3	90.0	6	biological_process	transcription
GO:0006355	42.6	68.8	8	biological_process	regulation of transcription DNA dependent
GO:0006357	39.9	67.2	9	biological_process	regulation of transcription from RNA polymerase II promoter
GO:0006364	97.5	81.0	8	biological_process	rRNA processing
GO:0006406	84.9	68.7	11	biological_process	mRNA export from nucleus
GO:0006412	65.4	81.1	7	biological_process	protein biosynthesis
GO:0006457	46.4	70.5	7	biological_process	protein folding
GO:0006461	44.3	68.4	6	biological_process	protein complex assembly
GO:0006470	64.7	72.7	8	biological_process	protein amino acid dephosphorylation
GO:0006487	66.7	80.2	10	biological_process	protein amino acid N linked glycosylation
GO:0006508	38.6	85.2	7	biological_process	proteolysis
GO:0006511	64.7	59.6	11	biological_process	ubiquitin dependent protein catabolism
GO:0006623	66.8	69.8	10	biological_process	protein targeting to vacuole
GO:0006629	49.3	91.7	5	biological_process	lipid metabolism
GO:0006810	48.5	89.6	5	biological_process	transport
GO:0006813	88.6	91.2	9	biological_process	potassium ion transport
GO:0006886	72.7	73.3	8	biological_process	intracellular protein transport
GO:0006888	70.8	56.1	9	biological_process	ER to Golgi vesicle mediated transport
GO:0006897	72.3	70.4	7	biological_process	endocytosis
GO:0006915	58.5	72.4	6	biological_process	apoptosis
GO:0006916	61.1	66.2	9	biological_process	anti apoptosis
GO:0006917	85.5	67.0	9	biological_process	induction of apoptosis
GO:0006928	49.7	81.7	5	biological_process	cell motility
GO:0006935	69.3	60.0	6	biological_process	chemotaxis
GO:0006936	91.7	90.6	4	biological_process	muscle contraction
GO:0006950	54.9	76.8	3	biological_process	response to stress
GO:0006954	54.5	68.6	6	biological_process	inflammatory response
GO:0006955	45.4	73.3	5	biological_process	immune response
GO:0006968	60.4	77.1	5	biological_process	cellular defense response
GO:0006979	74.5	63.4	6	biological_process	response to oxidative stress
GO:0007047	76.8	78.0	6	biological_process	cell wall organization and biogenesis
GO:0007050	88.3	65.4	8	biological_process	cell cycle arrest
GO:0007124	63.9	68.9	8	biological_process	pseudohyphal growth
GO:0007126	78.2	75.7	7	biological_process	meiosis
GO:0007131	81.1	69.7	9	biological_process	meiotic recombination
GO:0007155	18.7	93.8	3	biological_process	cell adhesion
GO:0007166	56.9	79.3	5	biological_process	cell surface receptor linked signal transduction
GO:0007186	59.1	83.0	6	biological_process	G protein coupled receptor protein signaling pathway
GO:0007242	53.2	81.0	5	biological_process	intracellular signaling cascade
GO:0007243	61.1	66.7	6	biological_process	protein kinase cascade
GO:0007268	51.6	83.1	6	biological_process	synaptic transmission
GO:0007283	84.6	72.5	6	biological_process	spermatogenesis
GO:0007399	31.9	80.1	4	biological_process	nervous system development
GO:0007417	48.8	92.9	5	biological_process	central nervous system development
GO:0007517	86.7	82.5	5	biological_process	muscle development
GO:0007601	78.9	80.4	7	biological_process	visual perception

GO:0008076	93.3	88.0	9	cellular_component	voltage gated potassium channel complex
GO:0008083	94.4	94.4	5	molecular_function	growth factor activity
GO:0008270	75.5	85.8	6	molecular_function	zinc ion binding
GO:0008284	43.3	83.3	6	biological_process	positive regulation of cell proliferation
GO:0008285	37.8	77.0	6	biological_process	negative regulation of cell proliferation
GO:0008380	83.5	88.5	8	biological_process	RNA splicing
GO:0008544	68.5	87.3	5	biological_process	epidermis development
GO:0008565	81.5	70.8	3	molecular_function	protein transporter activity
GO:0009060	91.7	80.7	8	biological_process	aerobic respiration
GO:0009653	45.8	89.7	3	biological_process	morphogenesis
GO:0009887	58.3	100.0	5	biological_process	organ morphogenesis
GO:0016192	68.0	64.0	6	biological_process	vesicle mediated transport
GO:0016481	76.6	66.1	8	biological_process	negative regulation of transcription
GO:0016563	48.8	85.5	3	molecular_function	transcriptional activator activity
GO:0016564	72.5	66.4	3	molecular_function	transcriptional repressor activity
GO:0016887	51.7	86.0	8	molecular_function	ATPase activity
GO:0019735	65.6	61.7	8	biological_process	antimicrobial humoral response (sensu Vertebrata)
GO:0030036	54.2	62.0	8	biological_process	actin cytoskeleton organization and biogenesis
GO:0030437	77.3	80.1	6	biological_process	sporulation (sensu Fungi)
GO:0045944	46.3	72.1	10	biological_process	positive regulation of transcription from RNA polymerase II promoter
GO:0051082	58.0	62.8	4	molecular_function	unfolded protein binding
